# *Rickettsia massiliae* and its public health significance across Palearctic and Oriental regions: a scoping review

**DOI:** 10.1186/s40249-026-01456-3

**Published:** 2026-07-13

**Authors:** Muhammad Kashif Obaid, Cai Yuchun, Shehla Shehla, Jin Luo, Takele Adugna Kassegn, Anwar Zaib Khan, Muhammad Rashid, Qiaoyun Ren, Guiquan Guan

**Affiliations:** 1 State Key Laboratory of Animal Disease Control and Prevention, College of Veterinary Medicine, Lanzhou University, Lanzhou Veterinary Research Institute, Chinese Academy of Agricultural Sciences, Lanzhou, Gansu China; 2 Key Laboratory of Veterinary Parasitology of Gansu Province, Gansu Province Research Center for Basic Disciplines of Pathogen Biology, Lanzhou, Gansu China; 3 National Institute of Parasitic Diseases, Chinese Center for Disease Control and Prevention (Chinese Center for Tropical Diseases Research), National Key Laboratory of Intelligent Tracking and Forecasting for Infectious Diseases, National Health Commission Key Laboratory of Parasite and Vector Biology, WHO Collaborating Centre for Tropical Diseases, National Center for International Research on Tropical Diseases, Ministry of Science and Technology, Shanghai, 200025 China; 4 Department of Zoology, Abdul Wali Khan University Mardan, Mardan, Khyber Pakhtunkhwa Pakistan; 5 College of Veterinary Medicine and Animal Sciences, University of Gondar, Post Office Box 196, Gondar, Ethiopia; 6 State Key Laboratory for Animal Disease Control and Prevention, Harbin Veterinary Research Institute, Chinese Academy of Agricultural Sciences, Harbin, China; 7 Department of Parasitology, Faculty of Veterinary and Animal Sciences, The Islamia University of Bahawalpur, Bahawalpur, Punjab Pakistan; 8 Hebei Key Laboratory of Animal Physiology, Biochemistry and Molecular Biology, Hebei Collaborative Innovation Center for Eco-Environment, Ministry of Education Key Laboratory of Molecular and Cellular Biology, College of Life Sciences, Hebei Normal University, Shijiazhuang, China

**Keywords:** Spotted fever group, *Rickettsia massiliae*, Human case, Palearctic and Oriental regions, Preventive measure

## Abstract

**Background:**

Arthropod including ticks, fleas, and lice have been found to be infected with *Rickettsia massiliae*, a pathogenic member of spotted fever group, causing rickettsiosis. This comprehensive scoping review summarize the known facts about its transmission, diagnostic-methods, phylogenetic position, human-case-reports, preventive-measures, and distribution in Palearctic and Oriental regions.

**Methods:**

Three main steps were followed to compile this study: explanation of objective(s), identification of relevant literature, and retrieval of data determined by inclusion and exclusion criteria. Various databases, including Science Direct, Web of Science, PubMed, Scopus, Cochrane, and Google Scholar were screened for relevant literature. Our objectives were to collect data regarding transmission method(s) of *R. massiliae*, identification-assay(s), phylogenetic position, clinical-reports globally, and distribution via possible vectors & host animals surviving in Palearctic and Oriental regions. Descriptive analysis has been conducted to plot the frequency graphs of reported numbers in different countries and hosts.

**Results:**

Findings presented that *R. massiliae* have been found across Palearctic and Oriental regions in 5 tick genera (*Rhipicephalus, Hyalomma, Haemaphysalis, Ixodes, Amblyomma,* and *Dermacentor*), 1 of louse (*Haematopinus*)*,* 1 of sheep ked (*Melophagus*)*,* and 1 of flea (*Archaeopsylla*), although their role as a vector(s) is still unknown. Dogs, sheep, cattle, and goats were recorded as epidemiologically important host animals for infected arthropods in 30, 23, 17, and 16 different studies, highlighting their role in its possible transmission. Additionally, *Rh. sanguineus* sensu lato (s.l) and *Rh. turanicus* were reported to infest the aforementioned animals, which is also being recognized as potential vector for its transmission. Highest number of *R. massiliae* reports (10) from different animals and vectors were recorded in China. Italy was recorded with highest (3) clinical cases of humans to-date. Moreover, transmission methods like transovarial via *Rh. turanicus* and horizontal/artificial-feeding via *Rh. sanguineus* s.l. and various diagnostic methods for *R. massiliae* have been documented.

**Conclusions:**

*R. massiliae* has been widely documented throughout Palearctic and Oriental regions, with human cases reported in about six countries. *Rh. sanguineus* and *Rh. turanicus* stands out the potential vectors, while further investigation into the implications of diverse range of arthropods in epidemiology of this bacterium.

**Graphical Abstract:**

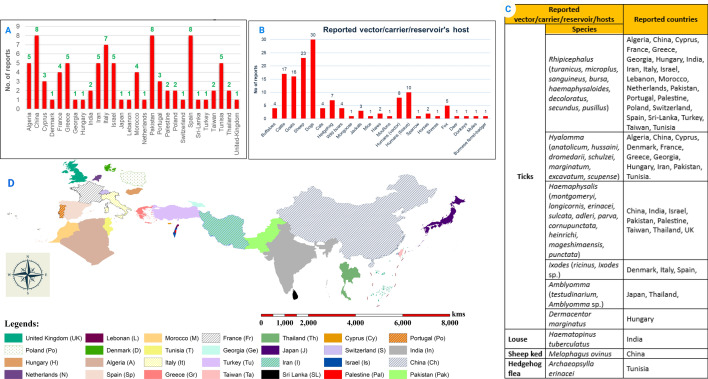

## Background

Rickettsiae are gram-negative bacteria that replicate entirely within cells and are named after Howard Ricketts, the American scientist who originally identified them as the cause of Rocky Mountain spotted fever in 1906 [1]. Rickettsial diseases are found globally, with several regions being endemic or hyper-endemic [2–4]. Rickettsiosis is an infectious disease caused by the obligate intracellular gram-negative bacteria, belonging to the order of rickettsiales, family Rickettsiaceae [5]. Rickettsiosis, caused by any other *Rickettsia* spp. except *R. massiliae*, is a significant contributor to morbidity and mortality in different regions of southeast Asian countries, and second most reported cause of non-malarial febrile illnesses, following dengue virus infection that is transmitted by mosquitoes in the region [6].

The family Rickettsiaceae includes the genera *Rickettsia* and *Orientia*. *Rickettsia* species are categorized according to differences in antigens and genomics. The taxonomy of this genus is currently growing and includes five distinct monophyletic groups: spotted fever group (SFG), transitional group, typhus group, *R. canadensis* group, and *R. bellii* group. Main disease-causing agents are prevalent worldwide and categorized mostly into SFG and Typhus groups [7–9]. Species in each of these groups vary in characteristics relating to their ecology, distribution, hosts, and pathogenicity [8]. All rickettsial species in these groups can potentially be propagated by ticks, fleas, lice, and mites in nature and can infect human beings [10–19]. The SFG rickettsial bacteria have a cosmopolitan distribution and are mainly propagated as well as transmitted to animals and humans via bites of approximately 198 infected arthropod vectors, which include species of ticks (*n* = 146), fleas (*n* = 24), mosquitoes (*n* = 15), mites (*n* = 6), lice (*n* = 4), keds (*n* = 2), and bugs (*n* = 1) [3, 4, 8, 13, 19]. Infected vectors transmit rickettsial bacteria through saliva while feeding on wounds or mucosal surfaces of host. The SFG species responsible for infectious illnesses are common in nature, with most human cases from the United States and Europe, with an estimated number of 66,133 human infections have been caused by SFG species worldwide from January 1906 to March 2021. The SFG rickettsiosis is becoming more widely recognized as a public health hazards globally, although its distribution and risk impact are not well defined [8, 13]. Although, insufficient information is available regarding the life cycles of majority of tick-borne (TB) Rickettsiae. Within natural vertebrate hosts, infections may lead to the development of rickettsemia, which enables noninfected ticks to get infected and maintains its natural life cycle [20]. During infection of SFG *Rickettsia*, propagation occurs via an infected arthropod vector, leading to the growth of *Rickettsia* in cells of endothelial layer. This process triggers inflammatory responses such as cytokine release and complement protein activation. *Rickettsia* growth and host immunological response cause the breakdown of blood vessels, leading to the distinctive maculopapular rash [21–23]. Although the bacteria mostly live in host's cytoplasm, certain bacteria leave via the luminal surface of vessel walls and enter into bloodstream [7].

The *R. massiliae* (Mtu1; Latin name of Marseille, France, where organism was first isolated) is an obligatory gram-negative intracellular organism. It flourishes in tissue cultures of L929, Vero cells, and human embryonic lung fibroblast cells. Gimenez-stained infected cells contain tiny, intracellular, rod-shaped, or diplobacilli bacteria that are somewhat smaller (0.3–0.4 by 0.6–1 µmol) than other SFG Rickettsiae. Electron microscopy of *R. massiliae* revealed an exterior slime layer and trilaminar cell wall. Bacteria develop unrestrictedly in cytoplasm of cells without a membrane boundary and are absent in nuclei of cells [24, 25]. *R. massiliae* is one of the SFG bacteria that show cosmopolitan distribution globally and has been detected in many types of arthropods and is transmitted by ticks in genus *Rhipicephalus*. *R. massiliae*, a novel rickettsial bacteria, was first discovered in hemolymph of *Rh. sanguineus* ticks infesting dogs in France in 1992. *Rh. sanguineus*, commonly known as brown dog tick, is a globally widespread tick species with significant medical and veterinary importance due to its role in transmitting various pathogens to dogs and, occasionally humans [26–28]. It can be found on dogs in both urban and rural areas, though it occasionally infests other hosts and is highly adapted to living inside human residences. This tick remains active throughout year, not only in tropical and subtropical regions but also in certain temperate areas. The ability of *Rh. sanguineus* to complete up to four generations per year depends on factors such as climate and host availability. High temperatures can cause ticks to attach and feed on humans and rabbits more quickly, suggesting an increased risk of human parasitism and transmission of zoonotic agents like *Rickettsia* spp. in areas with warmer or longer summers [27]. The taxonomy and systematics of *Rh. sanguineus* group have long been unclear, but recent research has clarified many aspects [29]. Additionally, *R. massiliae* was officially classified as a new species by Beati and Raoult in 1993 after confirming its distinctiveness through various identification methods [24, 25, 30]. In 1996, a variant strain of *R. massiliae* designated Bar-29, was isolated in a *Rh. sanguineus* tick from Spain while Bar-29 was characterized by phenotypic and genotypic analyses. The new isolates were confirmed to have the same profiles as *R. massiliae* through various methods that included micro-immunofluorescence serologic typing, sodium dodecyl sulfate-polyacrylamide gel electrophoresis, and polymerase chain reaction followed by restriction endonuclease fragment length polymorphism analysis. The profile of these isolates was distinct from other species of SFG and exhibited differentiable serotypes after identification via aforementioned assays [31]. *R. massiliae* Bar-29 has been detected in saliva of *Rh. turanicus* (within *Rh. sanguineus* group), providing evidence that bacteria could be transmitted through bite of this tick species [32]. Cardenosa et al. [33] concluded about pathogenicity of Bar-29 strain for humans. Additionally, it has been proved that *R. massiliae* has possibly transmitted by *Rh. sanguineus* s.l. tick species to dogs and to the patient’s family members which were documented positive with different levels of IgM and IgG for this pathogenic bacterium [34, 35].

Despite extensive literature on SFG Rickettsiae, a focused and comprehensive synthesis dedicated specifically to human pathogen, *R. massiliae*, has been lacking. This review fills that critical knowledge gap by providing a consolidated overview of key features of *R. massiliae*. The summarized information presented herein will aid in mitigation the impact of this emerging pathogen, and support the development of effective infection control strategies.

## Methods

**Fig. 1:**
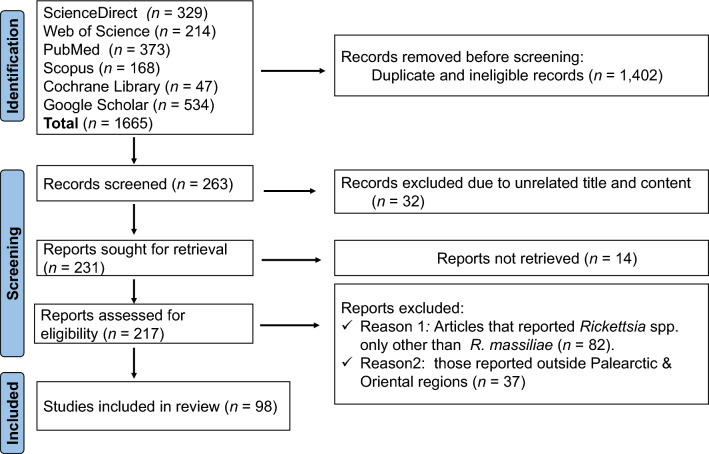
Method’s flow chart for article’s screening and processing

This scoping review began with the formation of a research team, including authors of this publication, who hold expertise in relevant topics. The investigators provided guidance on fundamental research question(s) to be explored, data search strategy, and criteria for inclusion and exclusion [36]. The research used a scoping review methodology in accordance with the Preferred Reporting Items for Systematic Reviews and Meta-Analyses extension for Scoping reviews (PRISMA-ScR), enabling a descriptive overview of the literature data as demonstrated in Fig. 1 [37, 38]. The study was conducted via a series of steps: (1) explanation of objective(s) and gap analysis; (2) identification and review of relevant literature; and (3) gathering of data determined by inclusion and exclusion criteria.

### Focused objective(s) and gaps analysis

To obtain literature-based data regarding various possible transmission methods, phylogenetic position (*ompA* gene), diagnostic methods, zoonotic and pathogenic impacts, possible treatment, and control measures of *R. massiliae* in targeted region were the main objectives of current study. As no comprehensive review, presenting each aforementioned parameter(s) associated with *R. massiliae*, was not currently available in literature. Hence, we planned to overcome this scientific gap to minimize the risks associated with this zoonotic bacterium.

### Literature data search strategy

To identify studies that align with our review objectives, a thorough computerized search strategy was devised for assembling all scientific papers that use English-language databases such as Science Direct, Web of Science, PubMed, Scopus, Cochrane, and Google Scholar to compile the appropriate scientific literature (Fig. 1). Various key terms, including SFG of *Rickettsia* and the detection of *R. massiliae* in various tick genera such as *Rhipicephalus, Haemaphysalis, Dermacentor, Ixodes*, *Hyalomma*, and *Amblyomma* were used in combinations in the aforementioned databases. Additionally, other terms were clinical cases of *R. massiliae* in humans, names of specific countries included in Palearctic & Oriental regions, microscopic, serological, and molecular detection of *R. massiliae* in cattle, buffaloes, sheep, goats, dogs, cats, foxes, jackals, wild boars, horses, donkeys, mules, hares, mice, mongooses, hedgehogs, sparrows, arthropods collected on vegetation, human beings, fleas, and louse were searched in different combinations in the aforementioned databases. Because of our main target, we intentionally employed the broader term "SFG *Rickettsia*" in our search strategy to ensure a comprehensive capture of literature. This approach mitigated the risk of omitting relevant studies that reported *R. massiliae* findings within general SFG surveillance or phylogenetic analyses but did not specify the species names including *R. massiliae,* in titles or keywords. Authors screened the obtained studies to determine if they contained individual data, removed duplicates, and excluded non-English literature after reviewing their titles and abstracts. Descriptive analysis has been conducted to plot the frequency graphs of the number of reports in different countries and different hosts.

### Inclusion and exclusion criteria

Specifically, this review is intended to provide an overview of the studies that have clearly mentioned any of the aforementioned focused question(s). Each country (included in Palearctic & Oriental regions) were searched to obtain the total reports till 4 June 2025, addressing any of the aforementioned objectives. Additionally, duplicate, unrelated title & content-based, published in other than our targeted regions, and reporting other *Rickettsia* spp., except *R. massiliae* studies were excluded. Moreover, we searched and included all the relevant literature regarding human infectious reports throughout the world (Fig. 1).

## Results and discussion

### Transmission of *R. massiliae*

**Fig. 2:**
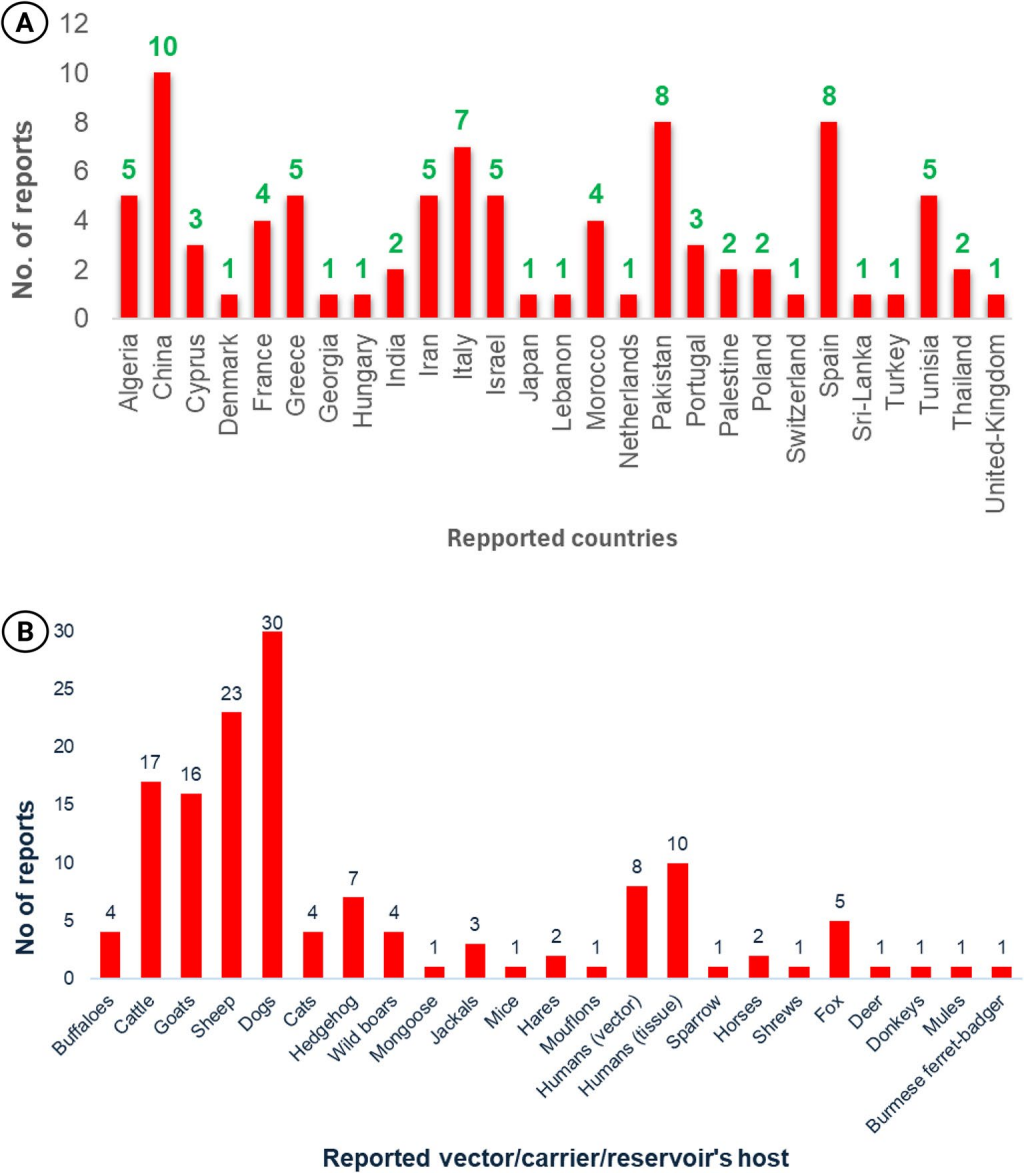
**A**–**B **: **A** Number of *Rickettsia massiliae* reports from each country (included in Palearctic and Oriental regions), **B** Number of identification reports from variety of host animals

Ticks acts as a vector/carrier/reservoir/host for vertical and horizontal transmission of Rickettsiae. This zoonotic bacteria has been detected globally in various tick species including *Rhipicephalus* (*turanicus, microplus, sanguineus, bursa, haemaphysaloides, decoloratus, secundus, pusillus*), *Hyalomma* (*anatolicum, hussaini, dromedarii, schulzei, marginatum, excavatum, scupense*), *Haemaphysalis* (*montgomeryi, longicornis, erinacei, sulcata, adleri, parva, cornupunctata, heinrichi, mageshimaensis, punctata*), *Ixodes* (*ricinus, Ixodes* sp.), *Amblyomma* (*testudinarium, Amblyomma* sp.), and *Dermacentor marginatus*. Additionally, it has been identified in *Haematopinus tuberculatus, Melophagus ovinus,* and *Archaeopsylla erinacei* as presented in Tables 1 and 2 [8, 80, 84, 88]. Proven vectors and infected arthropods have been marked clearly in Table 1. Further details regarding variety of potentially possible infected arthropods, their associated host animals, and number of reports from each country, included in Palearctic and Oriental regions, are presented in Fig. 2A–B.

**Table 1:** Published reports on *Rickettsia massiliae* detection in arthropods and the vertebrate animals on which arthropods were found, in Palearctic and Oriental regions

Country/year	Specimen type	Specimen sources	Serological (S)/molecular (M) evidences	Genetic marker (s)	Prevalence rate	References
Algeria/2001–2003	Rh. turanicus*	Cattle, goats, and hedgehog	M	*gltA* and* ompA*	Not known	[39]
	Rh. sanguineus*	Hedgehog				
Algeria/2010–2013	Rh. sanguineus*	Dogs and cats	M (qPCR)	*gltA*	24.4%	[40]
Algeria/2014	*Rh. bursa*	Cattle	M (RT-PCR)	*-*	79.7%	[41]
Algeria/2014–015	Rh. sanguineus*	Wild boars	M (qPCR)	*ompA*	18.5%	[42]
Algeria/2012	Rh. sanguineus*	Cattle, sheep, dogs, boars, Mongoose, and jackals	M	*ompA*	40.4%	[43]
	*Rh. bursa*					
China (northwest)	Rh. turanicus*	Sheep	M	*17-kDa, ompA, gltA, rrs, sca1,* and *ompB*	19.1%	[44]
China (Taklamakan desert)/2013–2014	Rh. turanicus*	Sheep	M	*ompA, gltA, rrs, geneD,* and *ompB*	3.3%	[45]
China/2015	*Hy. anatolicum*	Cattle	M	*ompA*	Not known	[46]
China	*Hae. longicornis*	Mice	M	*ompA*	Not known	[47]
China/2021–2022	*Melophagus ovinus*	Sheep	M	*17-kDa, rrs, gltA, sca1, ompA, ompB, gene D*	14.6%	[48]
China/2014–2016	Rh. turanicus*	Cattle and sheep	M	*17-kDa, rrs, gltA, sca1, ompA, ompB, gene D*	Not known	[49]
China/2017–2020	Rh. turanicus*	Pet dogs	M	*gltA* and* OmpB*	Not known	[50]
China/2016–2017	Rh. sanguineus*	Pet dogs	M	*17 kDa, rrs, gltA, ompA, ompB, geneD*	23.8%	[51]
China	Rh. sanguineus*	Dogs	M	*gltA* and* ompB*	2.8%	[52]
China	*Hae. mageshimaensis*	–	M	–	Not known	[53]
	*Hae. longicornis*					
Cyprus/1999–2006	Rh. turanicus*	Dogs, foxes, hares	M	*gltA* and* ompA*	10.3%	[54]
	*Rh. bursa*	Goats		*ompA*	2.7%	
	Rh. sanguineus*	Dogs			8.1%	
Cyprus	Rh. sanguineus*	Dogs and cats	M	*190-kDa*	5.4%	[55]
Cyprus/2002–2006	*Rh. bursa*	Mouflons	M	*ompA*	6.4%	[56]
Denmark/2011	*Ixodes* spp.	Dogs	M	*16S rRNA*	5.0%	[57]
France (southern)/2007	Rh. sanguineus*	Human (because human was detected positive for *Rickettsia* serologically)	S (IgG and IgM) and M	*ompA*	20.0%	[35]
France/2009	Rh. sanguineus*	Hedgehog	M (qPCR)	*gltA*	91.7%	[58]
France/2010	Rh. sanguineus*	House sparrow (*Passer domesticus*), dogs, horses, cat, vegetation, and human	M	*gltA* and* ompA*	28.8%	[59]
France	Rh. sanguineus*	Vegetation	M	monoclonal antibodies	-	[60]
Georgia/2012–2016	*Hy. marginatum*	Cattle, dogs, and vegetation	M and Sequencing	*gltA, ompA, ompB,* and *sca4*	1.2%	[61]
	Rh. sanguineus*	Dogs			1.2%	
Greece/2015	Rh. sanguineus*	Dogs	M	*ompB*	0.3%	[62]
	Rh. turanicus*				0.3%	
Greece/2009	*Rh. bursa*	Humans	M	*ompA*	12.5%	[63]
	Rh. turanicus*				42.8%	
Greece/2015–2016	*Rh. bursa*	Sheep	M	*16S rRNA*	–	[64]
Greece/1998–1999	Rh. turanicus*	Cattle, goats, and sheep	M	*gltA* and* ompA*	–	[65]
Greece/2014	Rh. sanguineus*	Dogs	M	*Atp, RecA, gltA, DnaA, DnaK, ompA* and* OmpB*	1.6%	[66]
Hungary/2011	*De. marginatus*	Shepherd dogs	RLB	–	7.7%	[67]
India/2017–2018	*Hae. bispinosa*	Cattle	M	*ompA*	Not known	[68]
India/2019–2020	Rh. sanguineus*	Shrews	M	*23S rRNA*	Not known	[69]
	*Haematopinus tuberculatus*	Buffaloes				
Iran/2017–2018	Rh. turanicus*	Cattle, goats, sheep, and dogs	M	*Rsp* and* gltA*	24.6%	[70]
	Rh. sanguineous*	Cattle, goats, and sheep			24.6%	
Iran/2018	*Hy. marginatum*	Goats and sheep	M	*gltA*	30.0%	[71]
	*Hy. anatolicum*					
	*Hy. dromedarii*					
	*Hy. schulzei*					
	*Rh. bursa*					
	Rh. turanicus*					
Iran/2019	Rh. sanguineus*	Dogs	M	*gltA, 17 kDa,* and ompA	Not Known	[72]
Iran	Rh. sanguineus*	Sheep and goats	M	*16S rRNA, gltA and ompA*	Not Known	[73]
Iran/2019–2020	Rh. sanguineus*	Dogs	M	*gltA*	2.0%	[74]
Israel/2010	Rh. turanicus*	Vegetation	M	*ompA*	1.7%	[75]
	Rh. sanguineus*	Dogs			0.3%	
	*Hae. erinacei*	Hedgehog			0.6%	
Israel/2002–2008	Rh. turanicus*	Vegetation	M	*ompA*	24.4%	[76]
	Rh. sanguineus*				9.2%	
Israel	Rh. sanguineus*	Vegetation	M	*16S rRNA*	Not known	[77]
Israel/2014	Rh. turanicus*	Vegetation	M	*gltA, ompA,* and* ompB*	Not known	[78]
	Rh. sanguineus*					
Israel/2014	Rh. turanicus*	Roe deer	M	*17 kDa,* and *ompA*	53.7%	[79]
Italy	Rh. sanguineus*	Foxes	M	*gltA*	7.1%	[80]
Italy/2014–2016	Rh. turanicus*	Humans	M	*23S rRNA, gltA,* and* ompA*	3.2%	[81]
		*Lepus europaeus* (Hare)				
	Rh. sanguineus*	Red fox				
Italy/2012–2013	Rh. sanguineus*	Dogs	M	*gltA, ompA,* and* ompB*		[82]
Italy/2011	Rh. turanicus*	Vegetation	M	*gltA, ompA,* and* ompB*	27.3%	[83]
	*Ix. ricinus*				17.4%	
Italy/2012–2013	Rh. sanguineus*	Humans	M	*gltA, ompA,* and* ompB*	Not known	[84]
	Rh. turanicus*					
Italy/2007–2010	*Ix. ricinus*	Vegetation	M	*gltA*	6.2%	[85]
Italy/2007	Rh. sanguineus*	Dogs	M	*gltA, ompA,* and* ompB*	Not known	[86]
	Rh. turanicus*	Cattle and goats				
Japan/1997–1999	*Amblyomma* sp.	Jackals	M	*gltA* and* ompA*	9.1%	[87]
Lebanon/2014	Rh. turanicus*	Ovine, bovine, and caprine	M	*ompA*	4.5%	[88]
Morocco/2002–2006	Rh. sanguineus*	Livestock and dogs	M	*12S rDNA, gltA,* and* ompA*	4.7%	[89]
Morocco/2018–2021	Rh. sanguineus*	Hedgehog	M	*gltA, ompA,* and* ompB*	5.6%	[90]
Morocco/2007	Rh. sanguineus*	Vegetation	M	*ompA*	26.7%	[91]
Morocco/2015–2020	Rh. sanguineus*	Foxes	M	*gltA, ompA,* and* ompB*	3.2%	[92]
Netherlands	Rh. sanguineus*	Sheep	M	*16S rRNA*	Not known	[93]
	Blood					
Pakistan/2017–2021	*Rh. haemaphysaloides*	Wild boars	M	*gltA* and* ompA*	8.3%	[94]
	Rh. turanicus*					
Pakistan	*Hy. anatolicum*	Cattle, buffaloes, Goats, and sheep	M	*16S rRNA* and* ompA*	0.2%	[95]
	*Rh. microplus*					
Pakistan	*Hae. sulcata*	Sheep and goats	M	*23S-5S ITS*	42.6%	[96]
	*Hy. anatolicum*					
	*Rh. microplus*					
	Rh. turanicus*					
	*Rh. haemaphysaloides*	Goats				
Pakistan/2017	*Hy. anatolicum*	Cattle and buffaloes	M	*23S-5S ITS*	1.7%	[97]
	*Hy. hussaini*					
Pakistan/2018–2019	*Rh. microplus*	Mules, horses, and donkeys	M	*gltA,* and* ompA*	2.4%	[98]
	*Rh. haemaphysaloides*					
	Rh. turanicus*					
	*Hy. anatolicum*					
Pakistan/2023–2024	*Rh. microplus*	Cattle, sheep and goats	M	*17 kDa, gltA,* and* ompA*	Not known	[99]
	Rh. sanguineus*					
Pakistan/2023	*Rh. haemaphysaloides*	Cattle	M	*gltA, ompA* and *ompB*	1.2%	[100]
	Rh. turanicus*					
	*Rh. microplus*	Goats				
	*Hae. cornupunctata*	Sheep				
Pakistan/2020–2022	*Hy. anatolicum*	Goats and sheep	M	*16S rRNA* and *gltA*	20.2%	[101]
	*Hy. excavatum*					
	*Hy. scupense*					
	*Hae. montgomeryi*					
	*Rh. decoloratus*					
	*Rh. haemaphysaloides*					
	*Rh. microplus*					
Palestine/2014	Rh. turanicus*	Dogs and sheep	M	*ompA, 16S rRNA* and* 17 kDa*	10.1%	[102]
	Rh. sanguineus*				7.4%	
	*Hae. adleri*				0.7%	
	*Hae. parva*				0.7%	
Palestine/2015	Rh. turanicus*	Sheep	M	*16S rRNA*	Not Known	[103]
Poland/2008–2009	Blood / serum	Humans	S	Specie specific antibodies	78.9%	[104]
Poland/2021	*Rh. secundus*	Vegetation	M	*gltA* and* ompA*	1.8%	[105]
Portugal/2021	Blood	Sheep	M	*ompB* and* ompA*	35.0%	[106]
	Rh. sanguineus*				62.0%	
Portugal/2002–2004	Rh. turanicus*	Birds	M	*gltA* and* ompA*	Not Known	[107]
Portugal/2011–2012	Rh. sanguineus*	Vegetation	M	*gltA*	9.0%	[108]
Spain/1997–2003	*Ixodes ricinus*	Humans	M	*gltA* and* ompA*	15.0%	[109]
	Rh. turanicus*					
	Rh. sanguineus*					
	*Rh. pusillus*					
Spain/2010–2016	Spleen	Dogs	M	*ITS2 gene*	1.6%	[110]
Spain	Blood	Red foxes	S	IgG-FITC antibody	50.3%	[111]
	Rh. sanguineus*		M	*ompA (qPCR), multiplex PCR*	78.1%	
Spain/2008	*Rh. pusillus*	Vegetation	M	*16S rRNA, ompB, atpA, dnaA, dnaK,* and *recA*	Not Known	[112]
Spain/1997–2001	Rh. sanguineus*	Humans	M	*gltA* and* ompA*	Not Known	[113]
Spain/2001–2005	Rh. sanguineus*	Humans and dogs	M	*gltA* and* ompA*	2.5%)	[114]
Spain	Rh. sanguineus*	Dogs	M	*16S rRNA* and *ITS2*	Not Known	[115]
Spain/2014–2016	Rh. sanguineus*	Wild boars	M	*16S rRNA* and *gltA*	45.2%	[116]
Sri Lanka	Rh. sanguineus*	Dogs	M	*ompA*	6.5%	[117]
		Cows				
Switzerland/1994–2001	Rh. sanguineus*	Dogs	M	*16S rDNA, gltA* and* ompA*	6.3%	[118]
Thailand/2008–2013	*Amb. Testudinarium*	Vegetations	M	*17-kDa gene*	Not known	[119]
Thailand/2015–2019	*Hae. heinrichi*	Burmese ferret-badger	M	*ompA, ompB,* and *sca4*	Not known	[120]
		Cattle				
Tunisia/2009	Rh. sanguineus*	Sheep	M	*23S-5S intergenic region* and* ompA*	0.7%	[121]
		Dogs				
Tunisia/2019–2020	*Hy. marginatum*	Cattle	M	*gltA, ompA,* and* ompB*	0.3%	[122]
	Rh. sanguineus*				10.67%	
Tunisia	Rh. sanguineus*	Goats and sheep	M	*gltA, ompA,* and* ompB*	Not known	[123]
	Rh. turanicus*					
Tunisia/2012–2014	Skin biopsies	Humans	M (qPCR)	*23S-5S rRNA*	63.7%	[124]
	Cutaneous swabs		RLB		40.6%	
Tunisia	Rh. sanguineus*	Hedgehog	M	*ompB*	Not known	[125]
	*Archaeopsylla erinaceid*					
Turkey/2016–2019	Rh. turanicus*	Dogs	M	*16S rRNA, gltA*, and *ompB*	1.8%	[126]
United Kingdom/2011	*Hae. punctata*	Vegetations	M	*16S rRNA*	2.0%	[127]

*Proven vectors, *Rh Rhipicephalus*, *Hy Hyalomma*, *Hae Haemaphysalis*, *De Dermacentor*, *S* serological, *M* molecular, *RLB* reverse line blot

year is representing the sampling time during specific study, while some studies were not having the exact sampling year

*gltA* Citrate synthase gene, *ompA* Outer membrane protein A, *ompB* Outer membrane protein B, *17-kDa* 17-kD antigen gene, *sca1* Surface cell antigen 1 gene, *geneD/sca4* Surface cell antigen 4 gene, *rRNA/rrs* Ribosomal RNA gene, *AtpA* ATP synthase F1 alpha subunit gene, *RecA* Recombination protein gene, *DnaA* Chromosomal replication initiation protein gene, *DnaK* Heat-shock protein 70 gene/chaperone protein, *ITS* Intergenic space gene

**Table 2:** Variety of vectors/carrier/reservoir/hosts and their reported countries

Reported vector/carrier/reservoir/hosts		Reported countries
**Ticks**	**Species**	
	*Rhipicephalus *(*turanicus, microplus, sanguineus, bursa, haemaphysaloides, decoloratus, secundus, pusillus*)	Algeria, China, Cyprus, France, Greece, Georgia, Hungary, India, Iran, Italy, Israel, Lebanon, Morocco, Netherlands, Pakistan, Portugal, Palestine, Poland, Switzerland, Spain, Sri-Lanka, Turkey, Tunisia
	*Hyalomma *(*anatolicum, hussaini, dromedarii, schulzei, marginatum, excavatum, scupense*)	Algeria, China, Cyprus, Denmark, France, Greece, Georgia, Hungary, Iran, Pakistan, Tunisia.
	*Haemaphysalis *(*montgomeryi, longicornis, erinacei, sulcata, adleri, parva, cornupunctata, heinrichi, mageshimaensis, punctata*)	China, India, Israel, Pakistan, Palestine, Thailand, UK
	*Ixodes *(*ricinus, Ixodes *sp.)	Denmark, Italy, Spain,
	*Dermacentor marginatus*	Japan, Thailand,
		Hungary
**Louse**	*Haematopinus tuberculatus*	India
**Sheep ked**	*Melophagus ovinus*	China
**Hedgehog flea**	*Archaeopsylla erinacei*	Tunisia

**Fig. 3:**
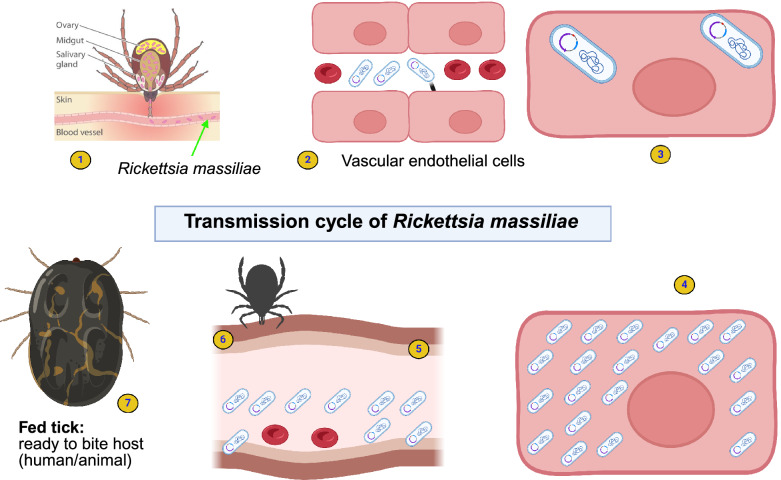
Putative mode of transmission of *Rickettsia massiliae* (not proven). Step 1: Biting of the tick to host animal for a blood meal and transfer of *R. massiliae* pathogen to the host, Step 2: Attachment of *R. massiliae* via adhesins (e.g., outer membrane protein B) to its host cells (vascular endothelial cells), Step 3: Engulfment of a bacterium by host cells, Step 4: The intracellular replication of *R. massiliae* by binary fission, Step 5: Destruction of vascular endothelial cells and release of *R. massiliae* into the blood vessels, that are ready to attach with other host cells (again infection starts), Step 6: Feeding of tick(s) for a blood meal from *R. massiliae* infected host animal, Step 7: Infected tick(s), which can bite another normal host (animal/human) and transfer the bacterium via saliva, hence the cycle starts back. Prepared via Biorender (Biorender (https://app.biorender.com) and according to Kim et al. [128]

These tick species are widely distributed and harbor various pathogens, including *R. massiliae,* affecting animals and humans [27]. These findings may suggest the existence of *R. massiliae* in host animals because this bacterium can be transmitted from tick(s) to host animals during blood feeding [23, 35, 111, 113] (Fig. 3).

Previous studies indicated that *R. massiliae* Bar-29 can be transmitted to vertebrate hosts via tick’s saliva during feeding. Furthermore, several other methods are also involved in the transmission of *Rickettsia* bacteria, which are discussed below.

Transovarial transmission is an efficient vertical transmission of rickettsial agents observed in many arthropod vectors, including ticks. It’s not a compulsory factor that such transmission will contribute to the amplification of TB pathogens, but only contribute to the maintenance of disease in surrounding environment [129]. More specifically, previous research has outlined the transovarial transmission of *R. massiliae* by identifying its presence in first-generation larvae of infected engorged *Rh. turanicus* ticks as well as by analyzing eggs from *R. massiliae* infected the same tick species by PCR. The observation that larvae and nymphs of second and third generation that fed on rabbits were positive for *R. massiliae*, suggests transovarial transmission. Infected female eggs, as well as larvae and nymphs throughout the developmental phases, exhibit a 100% transmission rate. Salivary transmission was detected in 80% of analyzed tick samples using PCR evaluation of saliva from the second generation of ticks from infected females [32].

This bacterium has been reported to be transmitted from ticks to blood via artificial feeding based on silicone membranes. In a previous study, *R. massiliae* infected *Rh. sanguineus* ticks were fed on bacteria-free bovine blood in an in vitro feeding system using a silicone membrane. Later, the blood samples were positive for the presence of *R. massiliae* via molecular techniques, and this was performed to validate that these ticks can act as vectors for *R. massiliae* [93].

Co-feeding/transsexual transmission of *R. massiliae* Bar-29 strain was evaluated by feeding *R. massiliae*-infected *Rh. turanicus* male ticks on a rabbit with non-infected *Rh. sanguineus* female ticks. After laying eggs, majority (69.2%) of female *Rh. sanguineus* were positive and confirmed via PCR for rickettsial *gltA*. Since, *Rh. turanicus* and *Rh. sanguineus* are closely related species; breeding between specimens of both species is conceivable (J. L. Camicas, personal communication) [32].

### Pathogenicity and human infection reports

To date, nine human cases of *R. massiliae* infection have been reported globally. First case of human infection was confirmed by molecular analysis of a 20-year frozen sample of a patient with an illness reminiscent of Mediterranean spotted fever (MSF) in Sicily (Italy) in 2006 [130]. The presence of *R. massiliae* was confirmed in serum samples of forest workers in Poland via indirect immunofluorescence assay (IFA), with subsequent molecular confirmation [104]. Serological techniques like direct IFA have been used to identify Rickettsiae in both humans and animals [35, 104, 111, 131, 132], however detecting these bacteria is challenging due to short durations of rickettsemia. Details regarding the nine human reports of *R. massiliae* are presented in Table 3.

**Table 3:** Detail of human cases regarding *Rickettsia massiliae*

Country	Gender	Age (y)	Year of infection/sample collection	Sign and symptoms	Diagnostic assay	Medication	Effectiveness of medication	Study
Sicilia (Italy)	M	45	6th June 1985/identified in 2005 as a and *R. massiliae* after 20 years sample storage	Fever, rash, eschar, hepatomegaly	Cell culture and PCR from blood	Tetracycline	Cured	[130]
France	M	25	24th May 2007/10th June 2007	Fever, eschar, rash, acute vision loss	PCR (cutaneous biopsy, aqueous humor	Doxycycline, Ofloxacin and Glucocorticoid (IV)	Ophthalmic sequelae	[133]
Argentina	F	56	Un-known/1st July 2005	Fever, purpuric rash eschar, pleural effusion	PCR (cutaneous biopsy)	Doxycycline (12 days)	Cured	[134]
Sicilia (Italy)	M	13	10th May 2012/un-known	Headache, fever, cervical lymphadenitis, eschar, hepatomegaly	PCR (cutaneous biopsy)	Doxycycline	Cured	[135]
Romania	M	76	June 2011–June 2012^a^	Fever, rash, conjunctivitis, myalgia, eschar	Western blot with cross absorption	Doxycycline (7 days)	Cured	[136]
	F	49		Fever, rash, myalgia, eschar	Indirect immunofluorescence assay	Ibuprofen, Doxycycline, ofloxacin (14 days)	Cured	
Greece	M	62	June 2013/July 2013	Fever, headache, myalgia, eschar, upper extremities rash	Indirect immunofluorescence assay	Ibuprofen, Doxycycline, ofloxacin (14 days)	Cured	[131]
Tunisia	Not known	Not known	2012–2014 from June to October^b^	Fever, rash, eschar	PCR on skin biopsy	Not known	Not known	[124]
Italy	F	61	Not known	Erythematous lesion on her right eyelid and latero-cervical satellite lymphadenopathy, occipital headache with painful neck, lowgrade fever, general malaise, asthenia and generalized joint pain	Biopsy and qPCR for swab of eyelid samples	Not known	Not known	[132]

*y* year, *M* Male, *F* Female

^a^Patients were examined during this time

^b^Study was conducted during this time, and no specific date of infection/sample collection is reported in both cases

The *R. massiliae* has been acknowledged as a newly identified etiological agent of MSF-like illnesses in humans and has an association with several tick species. The pathogenic role of *R. massiliae* was thought to be due to its sero-prevalence in humans. Some authors suggested that it may be the causal agent of certain cases of MSF [8]. To date, no case of *R. massiliae* infection has had a fatal outcome.

### Phylogenetic position

Molecular approach based on genetic variance at the individual base-pair level gives a much more direct pathway for measuring the genetic diversity between and within species of *Rickettsia* genus. The *gltA, ompA, ompB, 17-kDa, 190-kDa, 16S rRNA, 12S rDNA, 23S-5S ITS, ITS2, pgsA, Rsp, rrs, sca1,* and *sca4* genes are representative of different genomic functions and are believed to be active in most rickettsial species. Previous studies based on these molecular markers (Table 1) have been used for molecular detection and genetic analysis, providing sufficient and accurate information about evolutionary relationships of diverse *Rickettsia* species, specifically to characterize *R. massiliae* [52, 137, 138]. The *R. massiliae* group, specifically belonging to SFG *Rickettsia* and includes *R. massiliae*, *R. rhipicephali*, and *R. aeschlimannii* [139]. All about *R. massiliae* has been discussed earlier in this manuscript, however *R. rhipicephali* can infect ticks, and is generally considered a non-pathogenic agent especially for humans [140, 141] while *R. aeschlimannii* is recognized as an emerging human pathogen and is associated with ticks as a vector especially *Hy. marginatum* species [142], with cases reported in various regions of the world [143].

**Fig. 4:**
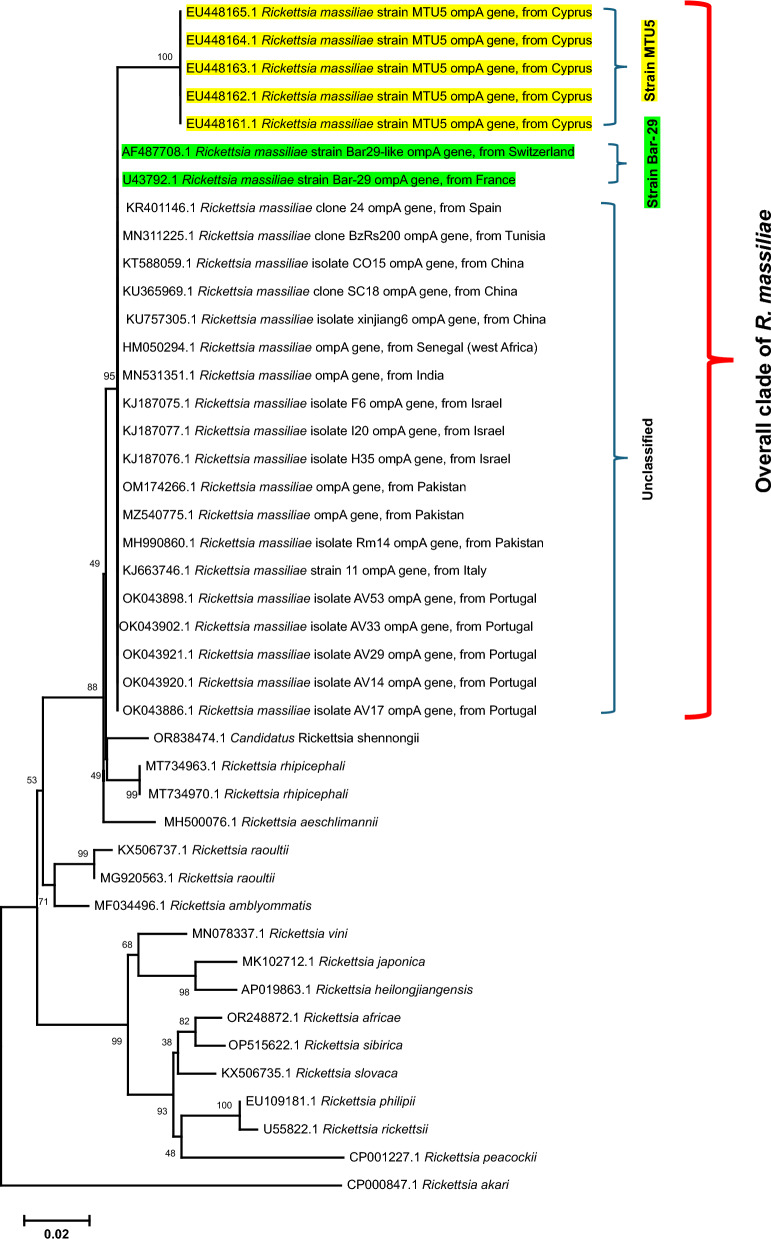
Constructed phylogenetic tree based on widely used genetic marker (*ompA*) sequences of *Rickettsia massiliae* using Minimum evolution method and p-distance model with 1000 bootstraps, representing that *R. massiliae* is making sister clade with *R*. *rhipicephali *and* R. aeschlimannii. *Three main groups including strain MTU5 (yellow), Bar-29 (green), and unclassified (no color) *R. massiliae* have been mentioned. *R. akari* (CP000847) was used as an outgroup (colour figure online)

The importance of comparing results obtained for multiple identification markers to test the reliability of organismal phylogeny has recently been highlighted. The method of choice to establish bacterial phylogeny (including *R. massiliae*) is the comparison of different gene sequences [93, 111, 144–146]. Although *R. massiliae* is one of the earliest diverging species of the SFG, its genome is very similar in terms of sequence and gene order to the genomes of other SFG [147]. The sequences (based on *ompA* gene) of *R. massiliae* from other countries appears highly similar with only limited divergence (Fig. 4). This close homology may reflect the limited resolution of the single *ompA* gene fragment employed, as well as the overall genetic conservation of *R. massiliae* across various regions including Cyprus, Switzerland, Israel, Portugal, Spain, Tunisia, China, Senegal, India, Italy, and Pakistan [44–46, 70, 71, 94, 96–98, 122].

### Diagnostic methods

In general, diagnosis of rickettsiosis is difficult because of the manifestations with non-specific clinical symptoms, such as fever, severe headache, skin rash, and general malaise, which is often misdiagnosed as other febrile illnesses or viral diseases. Confirmation of a rickettsial infection requires direct molecular detection because serological testing to detect antibodies potentially leads to a false negative result if serum-based testing occurs too early before antibodies are generated [148–150]. Moreover, proper testing requires expensive equipment, reagents, and skilled laboratory technicians. These aspects can complicate the ability of countries with constrained resources to test for *Rickettsia* infections. Cases of febrile illnesses are often over-diagnosed as malaria and later proven otherwise by more sensitive and specific PCR assays. Distinguishing *Rickettsia* from other pathogenic agents early allows for timely treatment and informs any necessary public health measures [151].

A laboratory diagnostic paradigm shift in rickettsial infections is evolving. The latest and accurate diagnostic assay should be owned to expand the diagnostic window toward early infection intervals of *R. massiliae*. Further advancements in field of *Rickettsia* spp. diagnostics are necessary in order to enhance the identification of diseases and epidemiological surveillance, as well as to get a deeper understanding of the dynamics of transmission. There has been a growing number of reports on the application of next-generation sequencing (NGS) and metagenomics for diagnosing *Rickettsia* spp. infection. NGS is used to analyze microbial communities, while metagenomics is particularly effective in identifying bacteria that are located inside cells, challenging to cultivate, or may not exhibit their characteristic molecular target (such as partial DNA sequences) [152]. Moreover, the editing technique, clustered regularly interspaced short palindromic repeats (CRISPR) based analyses will also facilitate comprehensive research on challenging bacterial species, specifically in the field of diagnosis [153]. Although numerous diagnostic assays have been previously developed for the identification of *R. massiliae* and its infection.

This bacterium has been identified via Gimenez staining of tick’s hemolymph. For the said purpose, 11 *Rh. turanicus* ticks were tested, including 5 females and 6 males. The fumigated ticks were used to cut their body parts (legs), and a drop of hemolymph was obtained that was stained with Gimenez for the successful examination of *R. massiliae* which were later validated via PCR assay [32].

The *R. massiliae* (strain MTU5) can be cultivated and cultured in L929 cells that were growing on Eagle’s Minimum Essential Medium (EMEM) (Biowhittaker, Cambrex Biosciences), treated with 2 mmol/L glutamine and 4% fetal calf serum (GIBCO, Invitrogen) in culture flasks and incubated at 32 °C for their optimum growth and division of cells. Molecular confirmation is provided by shotgun sequencing followed by Southern blot [147]. Another study stated that its diagnosis relies on its culturing in Vero cell line (African green monkey kidney epithelial cells) and observation of growth using Gimenez staining [111, 146]. *R. massiliae* has been successfully isolated molecularly via IFA and PCR in cultured tick’s cells [145]. The shell vial culture technique has been used for accurate identification of *R. massiliae* in tick cells. Rickettsial agents were observed by applying the Gimenez stain on tick cells on the 16th day of their culture, which was later confirmed and verified (on day 18th of culture) through PCR by the amplification of *gltA* and *ompA* genes [32].

PCR and sequencing methods are sensitive and rapid tools that are used to detect and identify Rickettsiae in blood, tissue, or skin biopsy specimens globally. Most reliable method used for the detection of *R. massiliae* involved amplification of specific segments of *R. massiliae* DNA by PCR. This involves the initial detection of species by screening all DNA samples by a real-time PCR assay targeting fragments of different genes, including *17-kDa, htrA*, *ompA*, *gltA*, *rrs*, *sca1*, *16S rRNA,12S rDNA,* and *ompB* [31, 76, 89, 93, 111, 145, 146]. Additionally, the PCR-restriction fragment length polymorphisms (RFLP) method enables the rapid identification of bacteria in amplified PCR products. Different endonucleases like RsaI and PstI were used for RFLP analyses for the identification and confirmation of *R. massiliae* and specifically, the Mtu5 isolate/strain of *R. massiliae* [154].

The *23S-5S* intergenic spacer gene can be molecularly amplified from the extracted DNA of ticks via PCR, followed by reverse line blot (RLB) hybridization for successful detection of *R. massiliae* with a sensitivity of 46.4% and specificity of 86.1%, using specific amino-oligonucleotide probes [67, 93, 121, 121].

The *R. massiliae* has been detected in serum, blood, spinal fluid, aqueous humor, and tissue specimens obtained from various animals by IFA, targeting IgG and IgM antibodies [34, 35, 104, 111, 131, 133]. Confirmation is subsequently provided by real-time PCR targeting the *gltA* gene [131]. The IFA was performed using human IgG against SFG *Rickettsia* provided in *Rickettsia* IFA IgG kit (Focus Diagnostics, Langenhagen Hannover, Germany) for the successful detection of *R. massiliae* [145]. *R. massiliae* Bar-29 has been successfully detected in saliva and feces of adult ticks through IFA, with successful amplification of *gltA* partial gene achieved by PCR [32].

Micro-immunofluorescence (MIF) is the ‘gold standard’ technique for the sero-diagnosis of MSF and is used as a reference technique in laboratories. This assay allows for simultaneous detection of multiple rickettsial agents in a single well because a single drop of diluted serum sample is reacted against many (usually nine) antigens simultaneously, which assist in differentiation of species [155]. This assay has been used for the detection of rickettsial agents, including *R. massiliae*, in Catalonian patients. Reactions to *R. massiliae* antigen can be very low but can be confirmed by specific reactions in western immunoblotting [33]. In a previous study, this bacterium has been detected using different assays, including IFA, MIF, SDS-PAGE, and western blot assay [31].

### Treatment of *R. massiliae* infection

Given the challenge of diagnosing any rickettsial infection during acute phase and need of prompt treatment for quick recovery and complication avoidance, it is advised to use anti-rickettsial medications, preferably tetracycline, as a preliminary prescription when rickettsiosis is suspected [130, 132, 149]. Typical treatment involves doxycycline at a dosage of 200 mg per day for a period of 3 to 14 days, depending on patient's clinical condition. Chloramphenicol and more recent macrolides are likely suitable substitutes for doxycycline [131, 156]. Additionally, previous studies have reported that other antibiotics, including ofloxacin and glucocorticoids have been successfully applied for the treatment of *R. massiliae* infections in humans (Table 2**)** [133]. Furthermore, this pathogenic bacterium has a natural resistance to rifampicin that is similar to the resistance displayed by *R. aeschlimannii* [130]. Therapeutic strategy is the same as recommended for all TB rickettsiosis [132, 157]; prompt doxycycline treatment should be initiated in case of clinical suspicion.

### Control/preventive measures

Additionally, there is a need to mitigate the potential vectors/reservoirs of *R. massiliae* using chemical acaricides, plant extracts, fungi, or vaccines [158–163]. It has been documented comprehensively that rickettsial infection rate has significantly increased from 33.3% in 2005 to 50.8% in 2015 (seasonally, especially from July to October) in Germany due to increased incidence of *Ixodes* ticks [164]. Early removal of ticks from their respective host animals (after attachment) could help in minimizing the risks of rickettsiosis. This is based on the detection of *R. massiliae* DNA as early as 8 h after the ticks were applied to host animals. These findings proved the early transmission of TB rickettsial DNA to host animals [93]. To prevent rickettsiosis, common risk environments should be avoided when traveling in endemic regions. These also include avoiding contact with rats, dogs, and domestic cattle, as well as bush plants that may be contaminated with ticks or mites. Specifically *Rh. sanguineus* s.l (competent proven vector) should be effectively eliminated via applications of different acaricides on its potential vectors (mainly dogs) as well as environments. The reinfestations after acaricides applications should be also minimized for a certain period of time [26, 165]. Unfortunately, resistance against various acaricides by this tick species has been reported globally, resulting in failure of its effusive control [166], although the mechanisms regarding development of resistance in this tick specie are poorly understood and demands further comprehensive mechanistic research. Additionally, molecular examination of human blood is also mandatory when relevant signs/symptoms are present, to check the presence of *R. massiliae*, so that control strategies could be developed to minimize the risks of human infections. For this purpose, recent advances in technology, such as next generation of sequencing and CRISPR-based gene editing, will facilitate further research on these obligate intracellular bacteria because these have been applied to identify many bacterial species, including those that pose challenges in terms of cultivation and manipulation, which presents a potential opportunity for the advancement of tools in *Rickettsia* species [153]. Moreover, the recombinase polymerase amplification (RPA) method in combination with a lateral flow (LF) test is recommended as a suitable approach for developing a straightforward, fast, highly sensitive, and specific method to detect *Rickettsia* species. This method would be particularly useful in areas with limited resources. The RPA technique involves three proteins: a recombinase, a polymerase, and a single-strand binding protein. It is capable of amplification within a time frame of 5–20 min at a constant temperature of approximately 37 °C. Furthermore, it has a detection limit of less than 10 copies per reaction. The amplified products are marked using a biotin-labelled reverse primer and a probe labelled with carboxyfluorescein (FAM) that is easily observable using the LF test [167].

### Limitations and future recommendations

Some unresolved questions regarding the confirmation of primary vertebrate host, reservoir hosts, or carriers for *R. massiliae* bacterium remained poorly defined because of the ambiguous role of vertebrate animals in its transmission and tick infestations. So future studies are recommended to comprehensively perform the xenodiagnoses based research and controlled experimental infections to move beyond the serosurveys for quantification of reservoir competence via amplification of *R. massiliae* bacterium. It is an important gap in literature, and more laboratory studies are needed to explore the vector capacity (transmission capability) of other arthropods, just like *Rh. turanicus* and *Rh. sanguineus* as proven vectors.

Moving forward, robust species-level confirmation via molecular methods in both typical and atypical SFG presentations is essential to definitively map the pathogenicity and true public health burden of this emerging agent. Specifically, the full spectrum of clinical signs from infection *R. massiliae* in people and animals should be documented and variations (if possible) in subspecies of *R. massiliae* should be addressed.

Similarly, future research outlining the concrete research approaches, including comparative genomics of humans vs tick-derived strains of *R. massiliae*, systematic febrile-illness surveillance, and refined animal models, should be performed to transform these uncertainties into a targeted research roadmap. We recommend that future research integrate detailed clinical reporting with whole-genome sequencing of patient-derived isolates should be performed, which is essential to determine if phylogenetic clades or specific virulence gene profiles correlate with disease severity. We believe that these approaches will resolve the unknown spectrum of *R. massiliae* pathogenicity (from endosymbiont-like relationships to genuine virulence) and will fulfil the existing knowledge gaps.

## Conclusions

Ticks associated rickettsial bacterium are growing day by day due to their variety of vectors and capacity to feed on various host animals. In Palearctic and Oriental regions, our study highlighted the geographical distribution of *R. massiliae* and its localization in all the infected arthropods as well as host animals, to recognize their existence and to know about their potential threats to the living community. Due to its potential threat for human beings, further investigations are required to minimize its zoonotic impacts. As most of the reported host animals are companions to humans beings and can be infested by variety of tick species belonging to *Rhipicephalus* genus, which increase the chances of their transmission to human beings, hence, *R. massiliae* is a human concern currently. Therefore, further comprehensive research studies are needed to mitigate the known vectors of *R. massiliae* in genus *Rhipicephalus*, so that negative impacts of *R. massiliae* could be minimized.

## Data Availability

Not applicable. As we have presented all the included literature data in the manuscript.

## Abbreviations

SFG: Spotted Fever Group
MSF: Mediterranean Spotted Fever
IFA: Immunofluorescence assay
NGS: Next-generation sequencing
CRISPR: Clustered regularly interspaced short palindromic repeats
LF: Lateral flow
RPA: Recombinase polymerase amplification
EMEM: Eagle’s minimum essential medium
RFLP: Restriction fragment length polymorphisms
RLB: Reverse line blot
MIF: Micro-immunofluorescence
PRISMA: Preferred reporting items for systematic reviews and meta-analyses
SDS-PAGE: Sodium dodecyl sulfate-polyacrylamide gel

## References

[1] Ricketts HT. The transmission of Rocky Mountain spotted fever by the bite of the wood-tick (*Dermacentor occidentalis*). JAMA J Am Med Assoc. 1906;XLVII:358. 10.1001/jama.1906.25210050042002j.

[2] Merhej V, Angelakis E, Socolovschi C, Raoult D. Genotyping, evolution and epidemiological findings of *Rickettsia* species. Infect Genet Evol. 2014;25:122–37. 10.1016/j.meegid.2014.03.014. 24662440 10.1016/j.meegid.2014.03.014

[3] Chikeka I, Dumler JS. Neglected bacterial zoonoses. Clin Microbiol Infect. 2015;21:404–15. 10.1016/j.cmi.2015.04.022. 25964152 10.1016/j.cmi.2015.04.022 PMC4466158

[4] Luce-Fedrow A, Mullins K, Kostik AP, St John HK, Jiang J, Richards AL. Strategies for detecting Rickettsiae and diagnosing rickettsial diseases. Future Microbiol. 2015;10:537–64. 10.2217/fmb.14.141. 25865193 10.2217/fmb.14.141

[5] Fournier P, Raoult D. Current knowledge on phylogeny and taxonomy of *Rickettsia* spp. Ann N Y Acad Sci. 2009;1166:1–11. 10.1111/j.1749-6632.2009.04528.x. 19538259 10.1111/j.1749-6632.2009.04528.x

[6] Acestor N, Cooksey R, Newton PN, Ménard D, Guerin PJ, Nakagawa J, et al. Mapping the aetiology of non-malarial febrile illness in Southeast Asia through a systematic review--terra incognita impairing treatment policies. PLoS ONE. 2012;7:e44269. 10.1371/journal.pone.0044269. 22970193 10.1371/journal.pone.0044269 PMC3435412

[7] Walker DH. Rickettsiae and rickettsial infections: the current state of knowledge. Clin Infect Dis. 2007;45(Supplement_1):S39-44. 10.1086/518145. 17582568 10.1086/518145

[8] Parola P, Paddock CD, Socolovschi C, Labruna MB, Mediannikov O, Kernif T, et al. Update on tick-borne rickettsioses around the world: a geographic approach. Clin Microbiol Rev. 2013;26:657–702. 10.1128/CMR.00032-13. 24092850 10.1128/CMR.00032-13 PMC3811236

[9] El Karkouri K, Ghigo E, Raoult D, Fournier P-E. Genomic evolution and adaptation of arthropod-associated *Rickettsia*. Sci Rep. 2022;12:3807. 10.1038/s41598-022-07725-z. 35264613 10.1038/s41598-022-07725-z PMC8907221

[10] Dobler G, Wölfel R. Typhus and Other Rickettsioses. Dtsch Ärztebl Int. 2009. 10.3238/arztebl.2009.0348. 19547738 10.3238/arztebl.2009.0348 PMC2689634

[11] Obaid MK, Shehla S, Guan G, Rashid M, Shams S. Genotyping of ticks: first molecular report of *Hyalomma asiaticum* and molecular detection of tick-borne bacteria in ticks and blood from Khyber Pakhtunkhwa, Pakistan. Front Cell Infect Microbiol. 2024;14:1346595. 10.3389/fcimb.2024.1346595. 38533383 10.3389/fcimb.2024.1346595 PMC10963394

[12] Obaid MK, Lan X, Ren Q, Zeb J, Luo J, Yang J, et al. Molecular insights into Rickettsiales in blood and ticks of two-humped camels at Gansu Province, China: with an accidental detection of *Colpodella* sp. Vet Microbiol. 2025;305:110528. 10.1016/j.vetmic.2025.110528. 40300414 10.1016/j.vetmic.2025.110528

[13] Zhang Y-Y, Sun Y-Q, Chen J-J, Teng A-Y, Wang T, Li H, et al. Mapping the global distribution of spotted fever group rickettsiae: a systematic review with modelling analysis. Lancet Digit Health. 2023;5:e5-15. 10.1016/S2589-7500(22)00212-6. 36424337 10.1016/S2589-7500(22)00212-6 PMC10039616

[14] Elelu N, Ola-Fadunsin SD, Bankole AA, Raji MA, Ogo NI, Cutler SJ. Prevalence of tick infestation and molecular characterization of spotted fever *Rickettsia massiliae* in *Rhipicephalus* species parasitizing domestic small ruminants in north-central Nigeria. PLoS ONE. 2022;17:e0263843. 10.1371/journal.pone.0263843. 35157723 10.1371/journal.pone.0263843 PMC8843212

[15] Lu M, Tian J, Wang W, Zhao H, Jiang H, Han J, et al. High diversity of *Rickettsia* spp., *Anaplasma* spp., and *Ehrlichia* spp. in ticks from Yunnan Province, Southwest China. Front Microbiol. 2022;13:1008110. 10.3389/fmicb.2022.1008110. 36312964 10.3389/fmicb.2022.1008110 PMC9606716

[16] Jin L, Huaijie J, Shuaiyang Z, Qiaoyun R, Qingli N, Jifei Y, et al. Microbial pathogen community in * Ornithodoros lahorensis * (Acari: Argasidae) in China. FASEB J. 2025;39:e70694. 10.1096/fj.202500105R. 40488750 10.1096/fj.202500105R

[17] Obaid MK, Ren Q, Zan X, Luo J, Zeb J, Rashid M, et al. Pathogen profiling and molecular evaluation along with in silico modeling of voltage‐gated sodium channel gene in Deltamethrin exposed *Haemaphysalis longicornis* ticks. FASEB J. 2025;39:e70907. 10.1096/fj.202501223RRR. 40779355 10.1096/fj.202501223RRR

[18] Obaid MK, Luo J, Zhao S, Tian Z, Ullah S, Zeb J, et al. Molecular profiling of ticks and associated pathogens: first report of *Rickettsia sibirica*, *Rickettsia slovaca*, and *Babesia microti* in ticks from Pakistan. Transbound Emerg Dis. 2025;2025:3157047. 10.1155/tbed/3157047. 41090112 10.1155/tbed/3157047 PMC12517992

[19] Portillo A, Santibáñez S, García-Álvarez L, Palomar AM, Oteo JA. Rickettsioses in Europe. Microbes Infect. 2015;17:834–8. 10.1016/j.micinf.2015.09.009. 26384814 10.1016/j.micinf.2015.09.009

[20] Parola P, Paddock CD, Raoult D. Tick-borne rickettsioses around the world: emerging diseases challenging old concepts. Clin Microbiol Rev. 2005;18:719–56. 10.1128/CMR.18.4.719-756.2005. 16223955 10.1128/CMR.18.4.719-756.2005 PMC1265907

[21] Blanton LS. The rickettsioses. Infect Dis Clin North Am. 2019;33:213–29. 10.1016/j.idc.2018.10.010. 30712763 10.1016/j.idc.2018.10.010 PMC6364315

[22] Mansueto P, Vitale G, Cascio A, Seidita A, Pepe I, Carroccio A, et al. New insight into immunity and immunopathology of rickettsial diseases. Clin Dev Immunol. 2012;2012:1–26. 10.1155/2012/967852. 10.1155/2012/967852 PMC3170826 21912565

[23] Sahni A, Fang R, Sahni SK, Walker DH. Pathogenesis of rickettsial diseases: pathogenic and immune mechanisms of an endotheliotropic infection. Annu Rev Pathol Mech Dis. 2019;14:127–52. 10.1146/annurev-pathmechdis-012418-012800. 10.1146/annurev-pathmechdis-012418-012800 PMC6505701 30148688

[24] Beati L, Finidori JP, Gilot B, Raoult D. Comparison of serologic typing, sodium dodecyl sulfate-polyacrylamide gel electrophoresis protein analysis, and genetic restriction fragment length polymorphism analysis for identification of rickettsiae: characterization of two new rickettsial strains. J Clin Microbiol. 1992;30:1922–30. 10.1128/jcm.30.8.1922-1930.1992. 1354221 10.1128/jcm.30.8.1922-1930.1992 PMC265417

[25] Beati L, Raoult D. *Rickettsia massiliae* sp. nov., a new spotted fever group *Rickettsia*. Int J Syst Bacteriol. 1993;43:839–40. 10.1099/00207713-43-4-839. 8240964 10.1099/00207713-43-4-839

[26] Dantas-Torres F. The brown dog tick, *Rhipicephalus sanguineus* (Latreille, 1806) (Acari: Ixodidae): from taxonomy to control. Vet Parasitol. 2008;152:173–85. 10.1016/j.vetpar.2007.12.030. 18280045 10.1016/j.vetpar.2007.12.030

[27] Dantas-Torres F. Biology and ecology of the brown dog tick, *Rhipicephalus sanguineus*. Parasit Vectors. 2010;3:26. 10.1186/1756-3305-3-26. 20377860 10.1186/1756-3305-3-26 PMC2857863

[28] Gray J, Dantas-Torres F, Estrada-Peña A, Levin M. Systematics and ecology of the brown dog tick, *Rhipicephalus sanguineus*. Ticks Tick-Borne Dis. 2013;4:171–80. 10.1016/j.ttbdis.2012.12.003. 23415851 10.1016/j.ttbdis.2012.12.003

[29] Dantas-Torres F, De Sousa-Paula LC, Otranto D. The *Rhipicephalus sanguineus* group: updated list of species, geographical distribution, and vector competence. Parasit Vectors. 2024;17:540. 10.1186/s13071-024-06572-3. 39731169 10.1186/s13071-024-06572-3 PMC11681662

[30] Diop A, Raoult D, Fournier P-E. Rickettsial genomics and the paradigm of genome reduction associated with increased virulence. Microbes Infect. 2018;20:401–9. 10.1016/j.micinf.2017.11.009. 29287988 10.1016/j.micinf.2017.11.009

[31] Beati L, Roux V, Ortuño A, Castella J, Porta FS, Raoult D. Phenotypic and genotypic characterization of spotted fever group Rickettsiae isolated from Catalan *Rhipicephalus sanguineus* ticks. J Clin Microbiol. 1996;34:2688–94. 10.1128/jcm.34.11.2688-2694.1996. 8897166 10.1128/jcm.34.11.2688-2694.1996 PMC229387

[32] Matsumoto K, Ogawa M, Brouqui P, Raoult D, Parola P. Transmission of *Rickettsia massiliae* in the tick, *Rhipicephalus turanicus*. Med Vet Entomol. 2005;19:263–70. 10.1111/j.1365-2915.2005.00569.x. 16134974 10.1111/j.1365-2915.2005.00569.x

[33] Cardeñosa N, Segura F, Raoult D. Serosurvey among Mediterranean spotted fever patients of a new spotted fever group rickettsial strain (Bar-29). Eur J Epidemiol. 2002;18:351–4. 10.1023/A:1023654400796. 10.1023/a:1023654400796 12803376

[34] Beeler E, Abramowicz KF, Zambrano ML, Sturgeon MM, Khalaf N, Hu R, et al. A focus of dogs and *Rickettsia massiliae*-infected *Rhipicephalus sanguineus* in California. Am J Trop Med Hyg. 2011;84:244–9. 10.4269/ajtmh.2011.10-0355. 21292893 10.4269/ajtmh.2011.10-0355 PMC3029176

[35] Renvoisé A, Delaunay P, Blanchouin E, Cannavo I, Cua E, Socolovschi C, et al. Urban family cluster of spotted fever rickettsiosis linked to *Rhipicephalus sanguineus* infected with *Rickettsia conorii* subsp. caspia and *Rickettsia massiliae*. Ticks Tick Borne Dis. 2012;3:389–92. 10.1016/j.ttbdis.2012.10.008. 23140893 10.1016/j.ttbdis.2012.10.008

[36] Levac D, Colquhoun H, O’Brien KK. Scoping studies: advancing the methodology. Implement Sci. 2010;5:69. 10.1186/1748-5908-5-69. 20854677 10.1186/1748-5908-5-69 PMC2954944

[37] Pham MT, Rajić A, Greig JD, Sargeant JM, Papadopoulos A, McEwen SA. A scoping review of scoping reviews: advancing the approach and enhancing the consistency. Res Synth Methods. 2014;5:371–85. 10.1002/jrsm.1123. 26052958 10.1002/jrsm.1123 PMC4491356

[38] Tricco AC, Lillie E, Zarin W, O’Brien KK, Colquhoun H, Levac D, et al. PRISMA extension for scoping reviews (PRISMA-ScR): checklist and explanation. Ann Intern Med. 2018;169:467–73. 10.7326/M18-0850. 30178033 10.7326/M18-0850

[39] Bitam I, Parola P, Matsumoto K, Rolain JM, Baziz B, Boubidi SC, et al. First molecular detection of *R. conorii*, *R. aeschlimannii*, and *R. massiliae* in ticks from Algeria. Ann N Y Acad Sci. 2006;1078:368–72. 10.1196/annals.1374.073. 17114743 10.1196/annals.1374.073

[40] Bessas A, Leulmi H, Bitam I, Zaidi S, Ait-Oudhia K, Raoult D, et al. Molecular evidence of vector-borne pathogens in dogs and cats and their ectoparasites in Algiers, Algeria. Comp Immunol Microbiol Infect Dis. 2016;45:23–8. 10.1016/j.cimid.2016.01.002. 27012917 10.1016/j.cimid.2016.01.002

[41] Dib L, Lafri I, Boucheikhchoukh M, Dendani Z, Bitam I, Benakhla A. Seasonal distribution of *Rickettsia* spp. in ticks in northeast Algeria. New Microbes New Infect. 2019;27:48–52. 10.1016/j.nmni.2018.10.008. 30622709 10.1016/j.nmni.2018.10.008 PMC6304374

[42] Zeroual F, Leulmi H, Saidani K, Benakhla A, Parola P, Bitam I. Detection of *Rickettsia* species in ticks Feeding on wild boar (Sus Scrofa Algira) in Algeria. 2024. 10.2139/ssrn.4973580. 10.1016/j.tvjl.2025.106468 41110825

[43] Leulmi H, Aouadi A, Bitam I, Bessas A, Benakhla A, Raoult D, et al. Detection of *Bartonella tamiae*, *Coxiella burnetii* and rickettsiae in arthropods and tissues from wild and domestic animals in northeastern Algeria. Parasit Vectors. 2016;9:27. 10.1186/s13071-016-1316-9. 26791781 10.1186/s13071-016-1316-9 PMC4721140

[44] Wei Q-Q, Guo L-P, Wang A-D, Mu L-M, Zhang K, Chen C-F, et al. The first detection of *Rickettsia aeschlimannii* and *Rickettsia massiliae* in *Rhipicephalus turanicus* ticks, in northwest China. Parasit Vectors. 2015;8:631. 10.1186/s13071-015-1242-2. 26652857 10.1186/s13071-015-1242-2 PMC4675064

[45] Guo L-P, Jiang S-H, Liu D, Wang S-W, Chen C-F, Wang Y-Z. Emerging spotted fever group rickettsiae in ticks, northwestern China. Ticks Tick Borne Dis. 2016;7:1146–50. 10.1016/j.ttbdis.2016.08.006. 27554852 10.1016/j.ttbdis.2016.08.006

[46] Yu P, Liu Z, Niu Q, Yang J, Abdallah MO, Chen Z, et al. Molecular evidence of tick-borne pathogens in *Hyalomma anatolicum* ticks infesting cattle in Xinjiang Uygur Autonomous Region, Northwestern China. Exp Appl Acarol. 2017;73:269–81. 10.1007/s10493-017-0162-6. 28875270 10.1007/s10493-017-0162-6

[47] Meng Z, Jiang L, Lu Q, Cheng S, Ye J, Zhan L. [Detection of co-infection with Lyme spirochetes and spotted fever group rickettsiae in a group of *Haemaphysalis longicornis*]. Zhonghua Liu Xing Bing Xue Za Zhi Zhonghua Liuxingbingxue Zazhi. 2008;29:1217–20. 19173967

[48] Li S, Zhang L, Li Z, Song H, Que Z, Zhao S, et al. Detection of *Rickettsia* spp. and *Anaplasma ovis* in *Melophagus ovinus* from southern Xinjiang, China. Med Vet Entomol. 2023;37:865–70. 10.1111/mve.12673. 37341627 10.1111/mve.12673

[49] Song S, Chen C, Yang M, Zhao S, Wang B, Hornok S, et al. Diversity of *Rickettsia* species in border regions of northwestern China. Parasit Vectors. 2018;11:634. 10.1186/s13071-018-3233-6. 30545379 10.1186/s13071-018-3233-6 PMC6293579

[50] Guo J, Song S, Cao S, Sun Z, Zhou Q, Deng X, et al. Molecular detection of zoonotic and veterinary pathogenic bacteria in pet dogs and their parasitizing ticks in Junggar Basin, North-Western China. Front Vet Sci. 2022;9:895140. 10.3389/fvets.2022.895140. 35898544 10.3389/fvets.2022.895140 PMC9311330

[51] Hazihan W, Dong Z, Guo L, Rizabek K, Askar D, Gulzhan K, et al. Molecular detection of spotted fever group rickettsiae in ticks parasitizing pet dogs in Shihezi City, northwestern China. Exp Appl Acarol. 2019;77:73–81. 10.1007/s10493-018-00337-1. 30649634 10.1007/s10493-018-00337-1 PMC6341051

[52] Chao L-L, Robinson M, Liang Y-F, Shih C-M. First detection and molecular identification of *Rickettsia massiliae*, a human pathogen, in *Rhipicephalus sanguineus* ticks collected from Southern Taiwan. PLoS Negl Trop Dis. 2022;16:e0010917. 10.1371/journal.pntd.0010917. 36367866 10.1371/journal.pntd.0010917 PMC9683588

[53] Liao J, Ho T, Liao K, Tu W, Lin Y. Comprehensive survey of tick infestations and pathogen detection in Taiwanese wildlife: uncovering public health risks. Zoonoses Public Health. 2025. 10.1111/zph.13227. 40457694 10.1111/zph.13227

[54] Chochlakis D, Ioannou I, Sandalakis V, Dimitriou T, Kassinis N, Papadopoulos B, et al. Spotted fever group Rickettsiae in Ticks in Cyprus. Microb Ecol. 2012;63:314–23. 10.1007/s00248-011-9926-4. 21833539 10.1007/s00248-011-9926-4

[55] Diakou A, Sofroniou D, Paoletti B, Tamvakis A, Kolencik S, Dimzas D, et al. Ticks, fleas, and harboured pathogens from dogs and cats in Cyprus. Pathogens. 2022;11:1403. 10.3390/pathogens11121403. 36558737 10.3390/pathogens11121403 PMC9786688

[56] Ioannou I, Sandalakis V, Kassinis N, Chochlakis D, Papadopoulos B, Loukaides F, et al. Tick-borne bacteria in mouflons and their ectoparasites in Cyprus. J Wildl Dis. 2011;47:300–6. 10.7589/0090-3558-47.2.300. 21441182 10.7589/0090-3558-47.2.300

[57] Stensvold CR, Al Marai D, Andersen LO, Krogfelt KA, Jensen JS, Larsen KS, et al. *Babesia* spp. and other pathogens in ticks recovered from domestic dogs in Denmark. Parasit Vectors. 2015;8:262. 10.1186/s13071-015-0843-0. 25951937 10.1186/s13071-015-0843-0 PMC4425907

[58] Marié J-L, Davoust B, Socolovschi C, Raoult D, Parola P. Molecular detection of rickettsial agents in ticks and fleas collected from a European hedgehog (*Erinaceus europaeus*) in Marseilles, France. Comp Immunol Microbiol Infect Dis. 2012;35:77–9. 10.1016/j.cimid.2011.11.005. 22169021 10.1016/j.cimid.2011.11.005

[59] Socolovschi C, Reynaud P, Kernif T, Raoult D, Parola P. Rickettsiae of spotted fever group, *Borrelia valaisiana*, and *Coxiella burnetii* in ticks on passerine birds and mammals from the Camargue in the south of France. Ticks Tick-Borne Dis. 2012;3:355–60. 10.1016/j.ttbdis.2012.10.019. 23141104 10.1016/j.ttbdis.2012.10.019

[60] Xu W, Raoult D. Production of monoclonal antibodies against *Rickettsia massiliae* and their use in antigenic and epidemiological studies. J Clin Microbiol. 1997;35:1715–21. 10.1128/jcm.35.7.1715-1721.1997. 9196180 10.1128/jcm.35.7.1715-1721.1997 PMC229828

[61] Sukhiashvili R, Zhgenti E, Khmaladze E, Burjanadze I, Imnadze P, Jiang J, et al. Identification and distribution of nine tick-borne spotted fever group Rickettsiae in the Country of Georgia. Ticks Tick-Borne Dis. 2020;11:101470. 10.1016/j.ttbdis.2020.101470. 32723640 10.1016/j.ttbdis.2020.101470

[62] Latrofa MS, Angelou A, Giannelli A, Annoscia G, Ravagnan S, Dantas-Torres F, et al. Ticks and associated pathogens in dogs from Greece. Parasit Vectors. 2017;10:301. 10.1186/s13071-017-2225-2. 28645329 10.1186/s13071-017-2225-2 PMC5481936

[63] Papa A, Xanthopoulou K, Kotriotsiou T, Papaioakim M, Sotiraki S, Chaligiannis I, et al. *Rickettsia* species in human-parasitizing ticks in Greece. Trans R Soc Trop Med Hyg. 2016;110:299–304. 10.1093/trstmh/trw022. 27198214 10.1093/trstmh/trw022 PMC4914872

[64] Papa A, Tsioka K, Daskou M-A, Minti F, Papadopoulou E, Melidou A, et al. Application of 16S rRNA next generation sequencing in ticks in Greece. Heliyon. 2020;6:e04542. 10.1016/j.heliyon.2020.e04542. 32760836 10.1016/j.heliyon.2020.e04542 PMC7393430

[65] Psaroulaki A, Ragiadakou D, Kouris G, Papadopoulos B, Chaniotis B, Tselentis Y. Ticks, tick‐borne rickettsiae, and *Coxiella burnetii* in the Greek Island of Cephalonia. Ann N Y Acad Sci. 2006;1078:389–99. 10.1196/annals.1374.077. 17114747 10.1196/annals.1374.077

[66] Moraga-Fernández A, Chaligiannis Ι, Cabezas-Cruz A, Papa A, Sotiraki S, De La Fuente J, et al. Molecular identification of spotted fever group *Rickettsia* in ticks collected from dogs and small ruminants in Greece. Exp Appl Acarol. 2019;78:421–30. 10.1007/s10493-019-00392-2. 31175472 10.1007/s10493-019-00392-2

[67] Hornok S, Fuente J, Horváth G, Fernández De Mera I, Wijnveld M, Tánczos B, et al. Molecular evidence of *Ehrlichia canis* and *Rickettsia massiliae* in ixodid ticks of carnivores from South Hungary. Acta Vet Hung. 2013;61:42–50. 10.1556/avet.2012.050. 23439290 10.1556/AVet.2012.050

[68] Krishnamoorthy P, Sudhagar S, Goudar AL, Jacob SS, Suresh KP. Molecular survey and phylogenetic analysis of tick-borne pathogens in ticks infesting cattle from two South Indian states. Vet Parasitol Reg Stud Rep. 2021;25:100595. 10.1016/j.vprsr.2021.100595. 10.1016/j.vprsr.2021.100595 34474788

[69] Candasamy S, Ayyanar E, Devaraju P, Kumar A, Zaman K, Bhaskar Mishra B, et al. Evidence on the prevalence of emerging and re‐emerging tick‐ and flea‐borne rickettsial agents in acute encephalitis syndrome endemic areas of Northeast Uttar Pradesh, India. Med Vet Entomol. 2024;38:23–37. 10.1111/mve.12694. 37736686 10.1111/mve.12694

[70] Ghasemi A, Latifian M, Esmaeili S, Naddaf SR, Mostafavi E. Molecular surveillance for *Rickettsia* spp. and *Bartonella* spp. in ticks from Northern Iran. PLoS ONE. 2022;17:e0278579. 10.1371/journal.pone.0278579. 36476750 10.1371/journal.pone.0278579 PMC9728842

[71] Afzalkhani A, Shayegh J, Jamali V, Malekzadeh P, Spotin A. Molecular detection and diversity of spotted fever group *Rickettsia* isolated from ticks in Iran. 2022. 10.21203/rs.3.rs-1324430/v1.

[72] Abdoli F, Mostafavi E, Esmaeili S, Rohani M. Molecular detection and identification of *Rickettsia* spp. in collected ticks from domestic animals in Southeastern of Iran. Comp Immunol Microbiol Infect Dis. 2022;85:101798. 10.1016/j.cimid.2022.101798. 35358741 10.1016/j.cimid.2022.101798

[73] Moravedji M, Latifian M, Rahravani M, Mostafavi E, Seyfi H, Mohammadi M, et al. Detection of various rickettsial species in ticks collected from small ruminants in Western Iran. Vector Borne Zoonotic Dis. 2024;24:730–7. 10.1089/vbz.2024.0014. 38856658 10.1089/vbz.2024.0014

[74] Mostafavi SM, Khalili M, Akhtardanesh B, Nourollahifard SR, Esmaeili S. *Rickettsia* spp. in *Rhipicephalus sanguineus* sensu lato ticks collected from stray dogs in Kerman city, Iran. Ticks Tick Borne Dis. 2022;13:101985. 10.1016/j.ttbdis.2022.101985. 35777305 10.1016/j.ttbdis.2022.101985

[75] Waner T, Keysary A, Eremeeva ME, Din AB, Mumcuoglu KY, King R, et al. *Rickettsia africae* and *Candidatus *Rickettsia barbariae in ticks in Israel. The American Society of Tropical Medicine and Hygiene. 2014;90:920–2. 10.4269/ajtmh.13-0697. 10.4269/ajtmh.13-0697 PMC4015588 24615133

[76] Harrus S, Perlman-Avrahami A, Mumcuoglu KY, Morick D, Baneth G. Molecular detection of *Rickettsia massiliae*, *Rickettsia sibirica mongolitimonae* and *Rickettsia conorii israelensis* in ticks from Israel. Clin Microbiol Infect. 2011;17:176–80. 10.1111/j.1469-0691.2010.03224.x. 20331680 10.1111/j.1469-0691.2010.03224.x

[77] Mumcuoglu KY, Arslan-Akveran G, Aydogdu S, Karasartova D, Koşar A, Savci U, et al. Pathogens in ticks collected in Israel: II. Bacteria and protozoa found in *Rhipicephalus sanguineus* sensu lato and *Rhipicephalus turanicus*. Ticks Tick-Borne Dis. 2022;13:101986. 10.1016/j.ttbdis.2022.101986. 35816829 10.1016/j.ttbdis.2022.101986

[78] Rose J, Nachum-Biala Y, Mumcuoglu KY, Alkhamis MA, Ben-Nun A, Lensky I, et al. Genetic characterization of spotted fever group Rickettsiae in questing ixodid ticks collected in Israel and environmental risk factors for their infection. Parasitology. 2017;144:1088–101. 10.1017/S0031182017000336. 28330517 10.1017/S0031182017000336

[79] Keysary A, Eremeeva ME, Leitner M, Din AB, Wikswo ME, Mumcuoglu KY, et al. Spotted fever group Rickettsiae in ticks collected from wild animals in Israel. The American Society of Tropical Medicine and Hygiene. 2011;85:919–23. 10.4269/ajtmh.2011.10-0623. 10.4269/ajtmh.2011.10-0623 PMC3205642 22049050

[80] Chisu V, Leulmi H, Masala G, Piredda M, Foxi C, Parola P. Detection of *Rickettsia hoogstraalii* , *Rickettsia helvetica* , *Rickettsia massiliae* , *Rickettsia slovaca* and *Rickettsia aeschlimannii* in ticks from Sardinia, Italy. Ticks Tick Borne Dis. 2017;8:347–52. 10.1016/j.ttbdis.2016.12.007. 28110915 10.1016/j.ttbdis.2016.12.007

[81] Pascucci I, Di Domenico M, Curini V, Cocco A, Averaimo D, D’Alterio N, et al. Diversity of *Rickettsia* in ticks collected in Abruzzi and Molise regions (Central Italy). Microorganisms. 2019;7:696. 10.3390/microorganisms7120696. 31847276 10.3390/microorganisms7120696 PMC6956140

[82] Scarpulla M, Barlozzari G, Marcario A, Salvato L, Blanda V, De Liberato C, et al. Molecular detection and characterization of spotted fever group Rickettsiae in ticks from Central Italy. Ticks Tick Borne Dis. 2016;7:1052–6. 10.1016/j.ttbdis.2016.06.003. 27365155 10.1016/j.ttbdis.2016.06.003

[83] Mancini F, Ciccozzi M, Lo Presti A, Cella E, Giovanetti M, Di Luca M, et al. Characterization of spotted fever group Rickettsiae in ticks from a city park of Rome. Italy Ann Ist Super Sanita. 2015;51:284–90. 10.4415/ANN_15_04_07. 26783214 10.4415/ANN_15_04_07

[84] Blanda V, Torina A, La Russa F, D’Agostino R, Randazzo K, Scimeca S, et al. A retrospective study of the characterization of *Rickettsia* species in ticks collected from humans. Ticks Tick Borne Dis. 2017;8:610–4. 10.1016/j.ttbdis.2017.04.005. 28457821 10.1016/j.ttbdis.2017.04.005

[85] Castro LR, Gabrielli S, Iori A, Cancrini G. Molecular detection of *Rickettsia*, *Borrelia*, and *Babesia *species in *Ixodes ricinus* sampled in northeastern, central, and insular areas of Italy. Exp Appl Acarol. 2015;66:443–52. 10.1007/s10493-015-9899-y. 25784072 10.1007/s10493-015-9899-y

[86] Mum A, Masala G, Tola S, Satta G, Fois F, Pirns P, et al. First direct detection of rickettsial pathogens and a new *Rickettsia*, “*Candidatus* Rickettsia barbariae”, in ticks from Sardinia, Italy. Clin Microbiol Infect. 2008;14:1028–33. 10.1111/j.1469-0691.2008.02082.x. 19040474 10.1111/j.1469-0691.2008.02082.x

[87] Satoh H, Motoi Y, Camer GA, Inokuma H, Izawa M, Kiyuuna T, et al. Characterization of spotted fever group Rickettsiae detected in dogs and ticks in Okinawa, Japan. Microbiol Immunol. 2002;46:257–63. 10.1111/j.1348-0421.2002.tb02694.x. 12061628 10.1111/j.1348-0421.2002.tb02694.x

[88] Fernández De Mera IG, Blanda V, Torina A, Dabaja MF, El Romeh A, Cabezas-Cruz A, et al. Identification and molecular characterization of spotted fever group rickettsiae in ticks collected from farm ruminants in Lebanon. Ticks Tick Borne Dis. 2018;9:104–8. 10.1016/j.ttbdis.2017.10.001. 29054546 10.1016/j.ttbdis.2017.10.001

[89] Sarih M, Socolovschi C, Boudebouch N, Hassar M, Raoult D, Parola P. Spotted fever group Rickettsiae in ticks, Morocco. Emerg Infect Dis. 2008;14:1067–73. 10.3201/eid1407.070096. 18598627 10.3201/eid1407.070096 PMC2600325

[90] Norte AC, Laghzaoui E-M, Guerreiro-Nunes A, El Mouden EH, Núncio MS, De Sousa R, et al. Molecular investigation of tick-borne pathogens from different regions of Morocco. Ticks Tick-borne Dis. 2024;15:102418. 10.1016/j.ttbdis.2024.102418. 39608244 10.1016/j.ttbdis.2024.102418

[91] Boudebouch N, Sarih M, Socolovschi C, Amarouch H, Hassar M, Raoult D, et al. Molecular survey for Spotted Fever Group Rickettsiae in ticks from Morocco. Clin Microbiol Infect. 2009;15:259–60. 10.1111/j.1469-0691.2008.02226.x. 19456813 10.1111/j.1469-0691.2008.02226.x

[92] Santos-Silva S, Santos N, Boratyński Z, Mesquita JR, Barradas PF. Diversity of *Rickettsia* spp. in ticks from wild mammals of Morocco and Mauritania. Ticks Tick Borne Dis. 2023;14:102235. 10.1016/j.ttbdis.2023.102235. 37531889 10.1016/j.ttbdis.2023.102235

[93] Olivieri E, Wijnveld M, Bonga M, Berger L, Manfredi MT, Veronesi F, et al. Transmission of *Rickettsia raoultii* and *Rickettsia massiliae* DNA by *Dermacentor reticulatus* and *Rhipicephalus sanguineus* (s.l.) ticks during artificial feeding. Parasit Vectors. 2018;11:494. 10.1186/s13071-018-3075-2. 30176918 10.1186/s13071-018-3075-2 PMC6122679

[94] Ali A, Shehla S, Zahid H, Ullah F, Zeb I, Ahmed H, et al. Molecular survey and spatial distribution of *Rickettsia* spp. in ticks infesting free-ranging wild animals in Pakistan (2017-2021). Pathogens. 2022;11:162. 10.3390/pathogens11020162. 35215108 10.3390/pathogens11020162 PMC8878123

[95] Rehman A, Conraths FJ, Sauter‐Louis C, Krücken J, Nijhof AM. Epidemiology of tick‐borne pathogens in the semi‐arid and the arid agro‐ecological zones of Punjab province, Pakistan. Transbound Emerg Dis. 2019;66:526–36. 10.1111/tbed.13059. 30383917 10.1111/tbed.13059

[96] Ghafar A, Khan A, Cabezas-Cruz A, Gauci CG, Niaz S, Ayaz S, et al. An assessment of the molecular diversity of ticks and tick-borne microorganisms of small ruminants in Pakistan. Microorganisms. 2020;8:1428. 10.3390/microorganisms8091428. 32957540 10.3390/microorganisms8091428 PMC7563897

[97] Ghafar A, Cabezas-Cruz A, Galon C, Obregon D, Gasser RB, Moutailler S, et al. Bovine ticks harbour a diverse array of microorganisms in Pakistan. Parasit Vectors. 2020;13:1. 10.1186/s13071-019-3862-4. 31900233 10.1186/s13071-019-3862-4 PMC6942265

[98] Ali A, Zahid H, Zeb I, Tufail M, Khan S, Haroon M, et al. Risk factors associated with tick infestations on equids in Khyber Pakhtunkhwa, Pakistan, with notes on *Rickettsia massiliae* detection. Parasit Vectors. 2021;14:363. 10.1186/s13071-021-04836-w. 34256806 10.1186/s13071-021-04836-w PMC8276440

[99] Jamil L, Li C, Wang Y, Jamil J, Tian W, Zhao D, et al. High diversity and low coinfections of pathogens in ticks from ruminants in Pakistan. Microorganisms. 2025;13:1276. 10.3390/microorganisms13061276. 40572164 10.3390/microorganisms13061276 PMC12195261

[100] Shehla S, Ullah F, Alouffi A, Almutairi MM, Khan Z, Tanaka T, et al. Association of SFG *Rickettsia massiliae* and *Candidatus Rickettsia shennongii* with different hard ticks infesting livestock hosts. Pathogens. 2023;12:1080. 10.3390/pathogens12091080. 37764888 10.3390/pathogens12091080 PMC10536372

[101] Zeb J, Song B, Khan MA, Senbill H, Aziz MU, Hussain S, et al. Genetic diversity of tick-borne zoonotic pathogens in ixodid ticks collected from small ruminants in Northern Pakistan. Infect Genet Evol. 2024;124:105663. 10.1016/j.meegid.2024.105663. 39208920 10.1016/j.meegid.2024.105663

[102] Ereqat S, Nasereddin A, Al-Jawabreh A, Azmi K, Harrus S, Mumcuoglu K, et al. Molecular detection and identification of spotted fever group rickettsiae in ticks collected from the West Bank, Palestinian Territories. PLoS Negl Trop Dis. 2016;10:e0004348. 10.1371/journal.pntd.0004348. 26771654 10.1371/journal.pntd.0004348 PMC4714870

[103] Ravi A, Ereqat S, Al-Jawabreh A, Abdeen Z, Abu Shamma O, Hall H, et al. Metagenomic profiling of ticks: identification of novel rickettsial genomes and detection of tick-borne canine parvovirus. PLoS Negl Trop Dis. 2019;13:e0006805. 10.1371/journal.pntd.0006805. 30640905 10.1371/journal.pntd.0006805 PMC6347332

[104] Podsiadły E, Chmielewski T, Karbowiak G, Kędra E, Tylewska-Wierzbanowska S. The occurrence of spotted fever rickettsioses and other tick-borne infections in forest workers in Poland. Vector Borne Zoonotic Dis. 2011;11:985–9. 10.1089/vbz.2010.0080. 21083370 10.1089/vbz.2010.0080

[105] Polsomboon Nelson S, Ergunay K, Bourke BP, Reinbold-Wasson DD, Caicedo-Quiroga L, Kirkitadze G, et al. Nanopore-based metagenomics reveal a new *Rickettsia *in Europe. Ticks Tick-Borne Dis. 2024;15:102305. 10.1016/j.ttbdis.2023.102305. 38150911 10.1016/j.ttbdis.2023.102305

[106] Mesquita JR, Santos-Silva S, De Sousa Moreira A, Baptista MB, Cruz R, Esteves F, et al. *Rickettsia massiliae* circulation in sheep and attached *Rhipicephalus sanguineus* in Central Portugal. Trop Anim Health Prod. 2022;54:199. 10.1007/s11250-022-03206-7. 35668327 10.1007/s11250-022-03206-7

[107] Santos-Silva MM, Sousa R, Santos AS, Melo P, Encarnação V, Bacellar F. Ticks parasitizing wild birds in Portugal: detection of *Rickettsia aeschlimannii*, *R. helvetica* and *R. massiliae*. Exp Appl Acarol. 2006;39:331–8. 10.1007/s10493-006-9008-3. 16897568 10.1007/s10493-006-9008-3

[108] Santos AS, De Bruin A, Veloso AR, Marques C, Pereira Da Fonseca I, De Sousa R, et al. Detection of *Anaplasma phagocytophilum*, *Candidatus Neoehrlichia *sp., *Coxiella burnetii* and *Rickettsia *spp. in questing ticks from a recreational park, Portugal. Ticks Tick Borne Dis. 2018;9:1555–64. 10.1016/j.ttbdis.2018.07.010. 30097348 10.1016/j.ttbdis.2018.07.010

[109] Fernández-Soto P, Pérez-Sánchez R, Díaz Martín V, Encinas-Grandes A, Álamo Sanz R. *Rickettsia massiliae* in ticks removed from humans in Castilla y León, Spain. Eur J Clin Microbiol Infect Dis. 2006;25:811–3. 10.1007/s10096-006-0217-9. 17061097 10.1007/s10096-006-0217-9

[110] Movilla R, Altet L, Serrano L, Tabar M-D, Roura X. Molecular detection of vector-borne pathogens in blood and splenic samples from dogs with splenic disease. Parasit Vectors. 2017;10:131. 10.1186/s13071-017-2074-z. 28285583 10.1186/s13071-017-2074-z PMC5346854

[111] Ortuño A, Sanfeliu I, Nogueras MM, Pons I, López-Claessens S, Castellà J, et al. Detection of *Rickettsia massiliae*/Bar-29 and *Rickettsia conorii* in red foxes (*Vulpes vulpes*) and their *Rhipicephalus sanguineus* complex ticks. Ticks Tick Borne Dis. 2018;9:629–31. 10.1016/j.ttbdis.2018.02.002. 29433817 10.1016/j.ttbdis.2018.02.002

[112] Fernández De Mera IG, Zivkovic Z, Bolaños M, Carranza C, Pérez-Arellano JL, Gutiérrez C, et al. *Rickettsia massiliae* in the Canary Islands. Emerg Infect Dis. 2009;15:1869–70. 10.3201/eid1511.090681. 22531111 10.3201/eid1511.090681 PMC2857243

[113] Merino FJ, Nebreda T, Luis Serrano J, Fernández-Soto P, Encinas A, Pérez-Sánchez R. Tick species and tick-borne infections identified in population from a rural area of Spain. Epidemiol Infect. 2005;133:943–9. 10.1017/S0950268805004061. 16181517 10.1017/S0950268805004061 PMC2870328

[114] Oteo JA, Portillo A, Santibáñez S, Pérez‐Martínez L, Blanco JR, Jiménez S, et al. Prevalence of Spotted Fever Group *Rickettsia* species detected in ticks in La Rioja, Spain. Ann N Y Acad Sci. 2006;1078:320–3. 10.1196/annals.1374.060. 17114730 10.1196/annals.1374.060

[115] Vila A, Estrada-Peña A, Altet L, Cusco A, Dandreano S, Francino O, et al. Endosymbionts carried by ticks feeding on dogs in Spain. Ticks Tick Borne Dis. 2019;10:848–52. 10.1016/j.ttbdis.2019.04.003. 31006611 10.1016/j.ttbdis.2019.04.003

[116] Castillo‐Contreras R, Magen L, Birtles R, Varela‐Castro L, Hall JL, Conejero C, et al. Ticks on wild boar in the metropolitan area of *Barcelona* (Spain) are infected with spotted fever group rickettsiae. Transbound Emerg Dis. 2022;69. 10.1111/tbed.14268. 10.1111/tbed.14268 34331835

[117] Dasch GA, Eremeeva ME, Zambrano ML, Premaratna R, Kularatne SAM, Jayanthe Rajapakse RPV. Molecular characterization of rickettsial agents in ticks (Acari: Ixodidae) from Sri Lanka. Am J Trop Med Hyg. 2022;106:1613–23. 10.4269/ajtmh.21-0995. 35405644 10.4269/ajtmh.21-0995 PMC9209928

[118] Bernasconi MV, Casati S, Péter O, Piffaretti J-C. Rhipicephalus ticks infected with *Rickettsia *and *Coxiella *in Southern Switzerland (Canton Ticino). Infect Genet Evol J Mol Epidemiol Evol Genet Infect Dis. 2002;2:111–20. 10.1016/s1567-1348(02)00092-8. 10.1016/s1567-1348(02)00092-8 12797987

[119] Nooroong P, Trinachartvanit W, Baimai V, Ahantarig A. Phylogenetic studies of bacteria (*Rickettsia, Coxiella, *and *Anaplasma*) in *Amblyomma* and *Dermacentor *ticks in Thailand and their co-infection. Ticks Tick-Borne Dis. 2018;9:963–71. 10.1016/j.ttbdis.2018.03.027. 29610046 10.1016/j.ttbdis.2018.03.027

[120] Hirunkanokpun S, Ahantarig A, Baimai V, Pramual P, Rakthong P, Trinachartvanit W. Spotted fever group R*ickettsia, Anaplasma* and *Coxiella*-like endosymbiont in *Haemaphysalis* ticks from mammals in Thailand. Vet Res Commun. 2022;46:1209–19. 10.1007/s11259-022-09980-x. 35945408 10.1007/s11259-022-09980-x PMC9684344

[121] Khrouf F, M’Ghirbi Y, Znazen A, Ben Jemaa M, Hammami A, Bouattour A. Detection of *Rickettsia *in *Rhipicephalus sanguineus* Ticks and *Ctenocephalides felis* Fleas from Southeastern Tunisia by Reverse Line Blot Assay. J Clin Microbiol. 2014;52:268–74. 10.1128/JCM.01925-13. 24226919 10.1128/JCM.01925-13 PMC3911462

[122] Kratou M, Belkahia H, Selmi R, Andolsi R, Dhibi M, Mhadhbi M, et al. Diversity and phylogeny of cattle ixodid ticks and associated spotted fever group *Rickettsia* spp. in Tunisia. Pathogens. 2023;12:552. 10.3390/pathogens12040552. 37111438 10.3390/pathogens12040552 PMC10146803

[123] Belkahia H, Selmi R, Zamiti S, Daaloul-Jedidi M, Messadi L, Ben SM. Zoonotic *Rickettsia *species in small ruminant ticks From Tunisia. Front Vet Sci. 2021;8:676896. 10.3389/fvets.2021.676896. 34124229 10.3389/fvets.2021.676896 PMC8187766

[124] Khrouf F, Sellami H, Elleuch E, Hattab Z, Ammari L, Khalfaoui M, et al. Molecular diagnosis of *Rickettsia *infection in patients from Tunisia. Ticks Tick-Borne Dis. 2016;7:653–6. 10.1016/j.ttbdis.2016.02.010. 26897395 10.1016/j.ttbdis.2016.02.010

[125] Balti G, Galon C, Derghal M, Souguir H, Guerbouj S, Rhim A, et al. *Atelerix algirus*, the North African hedgehog: suitable wild host for infected ticks and fleas and reservoir of vector-borne pathogens in Tunisia. Pathog Basel Switz. 2021;10:953. 10.3390/pathogens10080953. 10.3390/pathogens10080953 PMC8399139 34451417

[126] Demir S, Erkunt Alak S, Köseoğlu AE, Ün C, Nalçacı M, Can H. Molecular investigation of *Rickettsia *spp. and *Francisella tularensis* in ticks from three provinces of Turkey. Exp Appl Acarol. 2020;81:239–53. 10.1007/s10493-020-00498-y. 32394036 10.1007/s10493-020-00498-y

[127] Tijsse-Klasen E, Hansford KM, Jahfari S, Phipps P, Sprong H, Medlock JM. Spotted fever group rickettsiae in *Dermacentor reticulatus* and *Haemaphysalis punctata *ticks in the UK. Parasit Vectors. 2013;6:212. 10.1186/1756-3305-6-212. 23870197 10.1186/1756-3305-6-212 PMC3725166

[128] Kim HK. *Rickettsia* -host-tick interactions: knowledge advances and gaps. Infect Immun. 2022;90:e00621-21. 10.1128/iai.00621-21. 35993770 10.1128/iai.00621-21 PMC9476906

[129] Ravindran R, Hembram PK, Kumar GS, Kumar KGA, Deepa CK, Varghese A. Transovarial transmission of pathogenic protozoa and rickettsial organisms in ticks. Parasitol Res. 2023;122:691–704. 10.1007/s00436-023-07792-9. 36797442 10.1007/s00436-023-07792-9 PMC9936132

[130] Vitale G, Mansueto S, Rolain J-M, Raoult D. *Rickettsia massiliae* human isolation. Emerg Infect Dis. 2006;12:174–5. 10.3201/eid1201.050850. 16634183 10.3201/eid1201.050850 PMC3291392

[131] Chochlakis D, Bongiorni C, Partalis N, Tselentis Y, Psaroulaki A. Possible *Rickettsia massiliae* infection in Greece: an imported case. Jpn J Infect Dis. 2016;69:328–30. 10.7883/yoken.JJID.2015.195. 26370425 10.7883/yoken.JJID.2015.195

[132] Eldin C, Virgili G, Attard L, Edouard S, Viale P, Raoult D, et al. *Rickettsia massiliae* infection after a tick bite on the eyelid. Travel Med Infect Dis. 2018;26:66–8. 10.1016/j.tmaid.2018.08.002. 30114479 10.1016/j.tmaid.2018.08.002

[133] Parola P, Socolovschi C, Jeanjean L, Bitam I, Fournier P-E, Sotto A, et al. Warmer weather linked to tick attack and emergence of severe rickettsioses. PLoS Negl Trop Dis. 2008;2:e338. 10.1371/journal.pntd.0000338. 19015724 10.1371/journal.pntd.0000338 PMC2581602

[134] García-García JC, Portillo A, Núñez MJ, Santibáñez S, Castro B, Oteo JA. A patient from Argentina infected with *Rickettsia massiliae*. Am J Trop Med Hyg. 2010;82:691–2. 10.4269/ajtmh.2010.09-0662. 20348520 10.4269/ajtmh.2010.09-0662 PMC2844561

[135] Cascio A, Torina A, Valenzise M, Blanda V, Camarda N, Bombaci S, et al. Scalp eschar and neck lymphadenopathy caused by *Rickettsia massiliae*. Emerg Infect Dis. 2013;19. 10.3201/eid1905.121169. 10.3201/eid1905.121169 PMC3647502 23697545

[136] Zaharia M, Popescu CP, Florescu SA, Ceausu E, Raoult D, Parola P, et al. *Rickettsia massiliae* infection and SENLAT syndrome in Romania. Ticks Tick Borne Dis. 2016;7:759–62. 10.1016/j.ttbdis.2016.03.008. 27034192 10.1016/j.ttbdis.2016.03.008

[137] Yu X, Jin Y, Fan M, Xu G, Liu Q, Raoult D. Genotypic and antigenic identification of two new strains of spotted fever group rickettsiae isolated from China. J Clin Microbiol. 1993;31:83–8. 10.1128/jcm.31.1.83-88.1993. 8093253 10.1128/jcm.31.1.83-88.1993 PMC262626

[138] Roux V, Rydkina E, Eremeeva M, Raoult D. Citrate synthase gene comparison, a new tool for phylogenetic analysis, and its application for the rickettsiae. Int J Syst Bacteriol. 1997;47:252–61. 10.1099/00207713-47-2-252. 9103608 10.1099/00207713-47-2-252

[139] Sekeyova Z, Roux V, Raoult D. Phylogeny of *Rickettsia* spp. inferred by comparing sequences of “gene D”, which encodes an intracytoplasmic protein. Int J Syst Evol Microbiol. 2001;51:1353–60. 10.1099/00207713-51-4-1353. 11491333 10.1099/00207713-51-4-1353

[140] Labruna MB. Ecology of *Rickettsia* in South America. Ann N Y Acad Sci. 2009;1166:156–66. 10.1111/j.1749-6632.2009.04516.x. 19538276 10.1111/j.1749-6632.2009.04516.x

[141] Goel S, Kaura T, Bisht K, Kaur J, Mewara A, Lakshmi PVM, et al. First detection and genetic identification of *Rickettsia *spp*.* from ticks collected from rodents in north India. Indian J Med Microbiol. 2023;46:100475. 10.1016/j.ijmmb.2023.100475. 37688843 10.1016/j.ijmmb.2023.100475

[142] Sentausa E, El Karkouri K, Michelle C, Raoult D, Fournier P-E. Draft genome sequence of *Rickettsia aeschlimannii*, associated with *Hyalomma marginatum* ticks. Genome Announc. 2014;2:e00666-14. 10.1128/genomeA.00666-14. 25059861 10.1128/genomeA.00666-14 PMC4110219

[143] Raele DA, Cafiero MA. Rickettsial infection in the COVID-19 era: the correlation between the detection of *Rickettsia aeschlimannii* in ticks and storytelling photography of a presumable human rickettsiosis case. Microorganisms. 2023;11:2645. 10.3390/microorganisms11112645. 38004657 10.3390/microorganisms11112645 PMC10673559

[144] Olsen GJ, Woese CR. Ribosomal RNA: a key to phylogeny. FASEB J. 1993;7:113–23. 10.1096/fasebj.7.1.8422957. 8422957 10.1096/fasebj.7.1.8422957

[145] Cicuttin GL, Brambati DF, Rodríguez Eugui JI, Lebrero CG, De Salvo MN, Beltrán FJ, et al. Molecular characterization of *Rickettsia massiliae* and *Anaplasma platys* infecting *Rhipicephalus sanguineus* ticks and domestic dogs, Buenos Aires (Argentina). Ticks Tick-Borne Dis. 2014;5:484–8. 10.1016/j.ttbdis.2014.03.001. 24907186 10.1016/j.ttbdis.2014.03.001

[146] Segura F, Pons I, Sanfeliu I, Nogueras M-M. Shell-vial culture, coupled with real-time PCR, applied to *Rickettsia conorii* and *Rickettsia massiliae*-Bar-29 detection, improving the diagnosis of the Mediterranean spotted fever. Ticks Tick-Borne Dis. 2016;7:457–61. 10.1016/j.ttbdis.2016.01.008. 26830273 10.1016/j.ttbdis.2016.01.008

[147] Blanc G, Ogata H, Robert C, Audic S, Claverie J-M, Raoult D. Lateral gene transfer between obligate intracellular bacteria: evidence from the *Rickettsia massiliae* genome. Genome Res. 2007;17:1657–64. 10.1101/gr.6742107. 17916642 10.1101/gr.6742107 PMC2045148

[148] Kovácová E, Kazár J. Rickettsial diseases and their serological diagnosis. Clin Lab. 2000;46:239–45. 10853230

[149] Ericsson CD, Jensenius M, Fournier P-E, Raoult D. Rickettsioses and the international traveler. Clin Infect Dis. 2004;39:1493–9. 10.1086/425365. 15546086 10.1086/425365

[150] Paris DH, Dumler JS. State of the art of diagnosis of rickettsial diseases: the use of blood specimens for diagnosis of scrub typhus, spotted fever group rickettsiosis, and murine typhus. Curr Opin Infect Dis. 2016;29:433–9. 10.1097/QCO.0000000000000298. 27429138 10.1097/QCO.0000000000000298 PMC5029442

[151] Ghai RR, Thurber MI, El Bakry A, Chapman CA, Goldberg TL. Multi-method assessment of patients with febrile illness reveals over-diagnosis of malaria in rural Uganda. Malar J. 2016;15:460. 10.1186/s12936-016-1502-4. 27604542 10.1186/s12936-016-1502-4 PMC5015337

[152] Dulanto Chiang A, Dekker JP. From the pipeline to the bedside: Advances and challenges in clinical metagenomics. J Infect Dis. 2020;221(Supplement_3):S331–40. 10.1093/infdis/jiz151. 31538184 10.1093/infdis/jiz151 PMC7325616

[153] Vigouroux A, Bikard D. CRISPR tools to control gene expression in bacteria. Microbiol Mol Biol Rev. 2020;84:e00077-19. 10.1128/MMBR.00077-19. 32238445 10.1128/MMBR.00077-19 PMC7117552

[154] Eremeeva ME, Bosserman EA, Demma LJ, Zambrano ML, Blau DM, Dasch GA. Isolation and identification of *Rickettsia massiliae* from *Rhipicephalus sanguineus* ticks collected in Arizona. Appl Environ Microbiol. 2006;72:5569–77. 10.1128/AEM.00122-06. 16885311 10.1128/AEM.00122-06 PMC1538723

[155] Abdad MY, Abou Abdallah R, Fournier P-E, Stenos J, Vasoo S. A concise review of the epidemiology and diagnostics of rickettsioses: *Rickettsia* and *Orientia* spp. J Clin Microbiol. 2018;56:e01728-17. 10.1128/JCM.01728-17. 29769278 10.1128/JCM.01728-17 PMC6062794

[156] Raoult D. Antibiotic treatment of rickettsiosis, recent advances and current concepts. Eur J Epidemiol. 1991;7:276–81. 10.1007/BF00145677. 1884779 10.1007/BF00145677

[157] Delord M, Socolovschi C, Parola P. Rickettsioses and Q fever in travelers (2004–2013). Travel Med Infect Dis. 2014;12:443–58. 10.1016/j.tmaid.2014.08.006. 25262433 10.1016/j.tmaid.2014.08.006

[158] Jackson MA, Jaronski ST. Production of microsclerotia of the fungal entomopathogen *Metarhizium anisopliae* and their potential for use as a biocontrol agent for soil-inhabiting insects. Mycol Res. 2009;113:842–50. 10.1016/j.mycres.2009.03.004. 19358886 10.1016/j.mycres.2009.03.004

[159] De Souza Chagas AC, De Sena Oliveira MC, Giglioti R, Santana RCM, Bizzo HR, Gama PE, et al. Efficacy of 11 Brazilian essential oils on lethality of the cattle tick *Rhipicephalus (Boophilus) microplus*. Ticks Tick-Borne Dis. 2016;7:427–32. 10.1016/j.ttbdis.2016.01.001. 26867819 10.1016/j.ttbdis.2016.01.001

[160] Rego ROM, Trentelman JJA, Anguita J, Nijhof AM, Sprong H, Klempa B, et al. Counterattacking the tick bite: towards a rational design of anti-tick vaccines targeting pathogen transmission. Parasit Vectors. 2019;12:229. 10.1186/s13071-019-3468-x. 31088506 10.1186/s13071-019-3468-x PMC6518728

[161] Obaid MK, Almutairi MM, Alouffi A, Safi SZ, Tanaka T, Ali A. Assessment of cypermethrin and amitraz resistance and molecular profiling of voltage-gated sodium channel and octopamine tyramine genes of *Rhipicephalus microplus*. Front Cell Infect Microbiol. 2023;13:1176013. 10.3389/fcimb.2023.1176013. 37305408 10.3389/fcimb.2023.1176013 PMC10248163

[162] Obaid MK, Islam N, Alouffi A, Khan AZ, Da Silva Vaz I, Tanaka T, et al. Acaricides resistance in ticks: selection, diagnosis, mechanisms, and mitigation. Front Cell Infect Microbiol. 2022;12:941831. 10.3389/fcimb.2022.941831. 35873149 10.3389/fcimb.2022.941831 PMC9299439

[163] Obaid MK, Ren Q, Luo J, Wang J, Rashid M, Zeb J, et al. Evaluation of fipronil efficacy and first molecular report of gamma-aminobutyric acid (GABA) gated chloride channel gene of *Rhipicephalus microplus* ticks in China and Pakistan. Vet Parasitol. 2025;334:110407. 10.1016/j.vetpar.2025.110407. 39893705 10.1016/j.vetpar.2025.110407

[164] Blazejak K, Janecek E, Strube C. A 10-year surveillance of Rickettsiales (*Rickettsia* spp. and *Anaplasma phagocytophilum*) in the city of Hanover, Germany, reveals Rickettsia spp. as emerging pathogens in ticks. Parasit Vectors. 2017;10:588. 10.1186/s13071-017-2537-2. 29179774 10.1186/s13071-017-2537-2 PMC5704456

[165] Dantas-Torres F, Figueredo LA, Brandão-Filho SP. *Rhipicephalus sanguineus* (Acari: Ixodidae), the brown dog tick, parasitizing humans in Brazil. Rev Soc Bras Med Trop. 2006;39:64–7. 10.1590/S0037-86822006000100012. 16501769 10.1590/s0037-86822006000100012

[166] Estrada-Pena A. tude de la résistance de la tique brune du chien, *Rhipicephalus sanguineus* aux acaricides. 2005.

[167] Qi Y, Shao Y, Rao J, Shen W, Yin Q, Li X, et al. Development of a rapid and visual detection method for *Rickettsia rickettsii* combining recombinase polymerase assay with lateral flow test. PLoS ONE. 2018;13:e0207811. 10.1371/journal.pone.0207811. 30475889 10.1371/journal.pone.0207811 PMC6257923

